# TGF-β and TNF-α interaction promotes the expression of MMP-9 through H3K36 dimethylation: implications in breast cancer metastasis

**DOI:** 10.3389/fimmu.2024.1430187

**Published:** 2024-09-16

**Authors:** Shihab Kochumon, Amnah Al-Sayyar, Texy Jacob, Fatemah Bahman, Nadeem Akhter, Ajit Wilson, Sardar Sindhu, Yusuf A. Hannun, Rasheed Ahmad, Fahd Al-Mulla

**Affiliations:** ^1^ Immunology and Microbiology Department, Dasman Diabetes Institute, Dasman, Kuwait; ^2^ Centre d’Immunologie de Marseille-Luminy, Aix Marseille Université, Inserm, Marseille, France; ^3^ Stony Brook Cancer Center, Stony Brook University, Stony Brook, NY, United States; ^4^ Translational Research, Dasman Diabetes Institute, Dasman, Kuwait

**Keywords:** histone methylation, H3K36me2, MMP-9, Smad2/3, TNF-α, TGF-β, Breast cancer

## Abstract

Increased MMP-9 expression in the tumor microenvironment (TME) plays a crucial role in the extracellular matrix remodeling to facilitate cancer invasion and metastasis. However, the mechanism of MMP-9 upregulation in TME remains elusive. Since TGF-β and TNF-α levels are elevated in TME, we asked whether these two agents interacted to induce/augment MMP-9 expression. Using a well-established MDA-MB-231 breast cancer model, we found that the synergy between TGF-β and TNF-α led to MMP-9 upregulation at the transcriptional and translational levels, compared to treatments with each agent alone. Our *in vitro* findings are corroborated by co-expression of elevated MMP-9 with TGF-β and TNF-α in human breast cancer tissues. Mechanistically, we found that the MMP-9 upregulation driven by TGF-β/TNF-α cooperativity was attenuated by selective inhibition of the TGF-βRI/Smad3 pathway. Comparable outcomes were observed upon inhibition of TGF-β-induced phosphorylation of Smad2/3 and p38. As expected, the cells defective in Smad2/3 or p38-mediated signaling did not exhibit this synergistic induction of MMP-9. Importantly, the inhibition of histone methylation but not acetylation dampened the synergistic MMP-9 expression. Histone modification profiling further identified the H3K36me2 as an epigenetic regulatory mark of this synergy. Moreover, TGF-β/TNF-α co-stimulation led to increased levels of the transcriptionally permissive dimethylation mark at H3K36 in the MMP-9 promoter. Comparable outcomes were noted in cells deficient in NSD2 histone methyltransferase. In conclusion, our findings support a cooperativity model in which TGF-β could amplify the TNF-α-mediated MMP-9 production via chromatin remodeling and facilitate breast cancer invasion and metastasis.

## Graphical Abstract

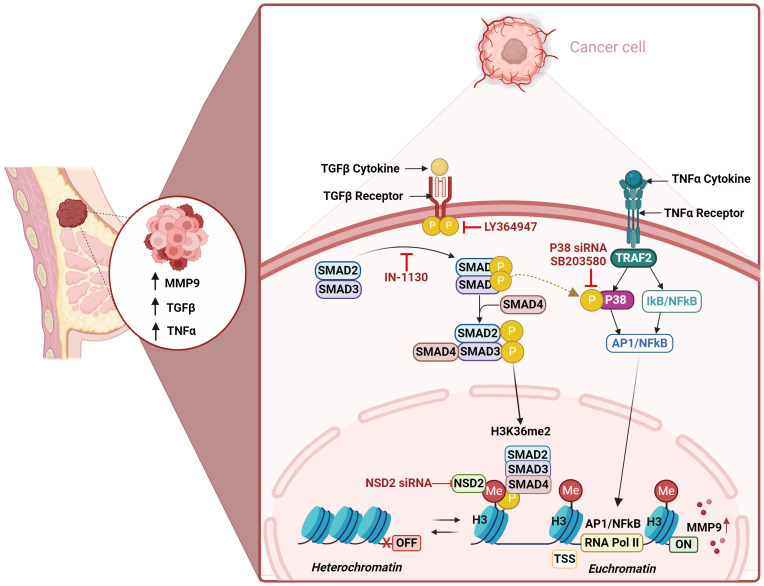

## 1 Introduction

The burden of breast cancer (BC) incidence and mortality are rapidly growing worldwide, especially among women. In 2020, 2.3 million females were diagnosed with BC with a mortality rate of 29.7% and the incidence rates are expected to increase by 40% in 2040 (1, 2). The increase in BC incidence rates is linked with either inherent risk factors (i.e. age, ethnicity, and genetics) or extrinsic factors (i.e. lifestyle, diet, metabolic and hormonal therapies) all of which usually affect tumor prognosis and influence cancer treatment therapies (3). In addition, recent studies indicated that genetic alterations and epigenetic modifications are the primary initiators of BC development and metastasis (4, 5). One known characteristic of BC is that it is a heterogenous disease, in which not only it consists of malignant cells, but also a diverse population of other cells (i.e. immune cells and stromal cells) that surround the extracellular matrix (ECM) forming the tumor microenvironment (5, 6). At the early stages of tumorigenesis, the cancer cells inhibit the cytotoxic function of the immune cells within the TME, allowing the formation of a blood vessel network that penetrates the tumor mass through angiogenesis, creating an environment that promotes cancer invasion and metastasis (7). Thus, alterations in the pathophysiology of TME affect the secretory activity of its components. This directly induces the production of proinflammatory cytokines (Tumor necrosis factor–α: TNF-α, Interleukin-1 beta (IL-1β), chemokines (chemokine (C-X-C motif) ligand 1 (CXCL1), CXCL10), and growth factors (transforming growth factor-β: TGFβ), that promote tumor activity and affect patient survival (7–9).

Matrix Metalloproteinases (MMPs) are a family of extracellular proteases that play an important role in facilitating cancer invasion and metastasis through the degradation of the ECM basement membrane causing DNA damage and genetic instability (10). Matrix metalloproteinase-9 (MMP-9) or gelatinase B has been directly linked with tumor invasion and metastasis due to its ability to degrade collagen type IV in the ECM supporting the formation of TME (11). Several studies highlighted that elevated level of MMP-9 is associated with higher tumor grade and reduced survival rate in patients with BC (10–12). These studies significantly addressed the role of MMP-9 in BC progression, making it an attractive target for antimetastatic therapies. Since MMP-9 is associated with cancer invasion and metastasis, regulation of MMP-9 is an important therapeutic approach for combating cancer invasion and metastasis. Despite the recognized importance of MMP-9 in breast cancer progression, the regulatory mechanisms governing its expression within the TME remain incompletely understood. Increased dysregulation of cytokines (TNF-α, TGF-β, IL-1β, etc.) along with increased expression of MMP-9 are present in TME.

TNF-α, is a proinflammatory cytokine that is involved in apoptosis, inflammation and immunity (13). The involvement of TNF-α in BC progression is well documented as its upregulation is significantly associated with BC recurrence (14, 15). Eftekhari et al., investigated the inflammatory composition of TME in human BC biopsies and found that TNF-α levels increase in parallel to the disease stage (16). This was evident *in vitro*, as high TNF-α levels induced BC cells migration and proliferation which was accompanied by increased MMP-9 secretion (17). Another study also reported the upregulation of TNF-α induces MMP-9 expression, and this effect was reversed through the inhibition of Sirtuin 6 (SIRT6) gene and mitogen-activated protein kinase (MAPK) phosphorylation (18). This indicates that both TNF-α and MMP-9 contribute to the dysregulation of the TME and promote metastasis.

TGF-β, is a polypeptide growth inhibitor that is related to molecules with wide range of regulatory activities and is involved in many developmental and physiological processes such as embryogenesis, immune responses, pro-inflammatory and anti-inflammatory processes (19). Roberts and Wakefield stated that the perturbations of the TME combined with changes in the genetic and epigenetic context of the tumor cell alters TGF-β activity to either promote or suppress tumor formation (20). For example, at the early stages of tumorigenesis, TGF-β inhibits the occurrence of tumors, however, in middle-late stages of the disease, TGF-β participates in malignant progression and tumor formation (21). A clinical study indicated that 81% of BC patients exhibited high TGF-β1 levels in plasma and upon tumor removal, TGF-β1 levels were normalized (22). On the other hand, immunohistochemistry assessment of human BC tissues showed significant increase in TGF-β1 expression, and it was mainly localized at the tumor edges and lymph node, indicating TGFβ1 role in promoting invasion and metastasis (23). Furthermore, the increase in cell malignancy was significantly associated with the overexpression of MMP-9 through the activation of TGF-β/Smad signaling pathway (24). Sun et al., found that resveratrol inhibits MDA-MB-231 cells migration by reversing TGF-β tumorigenic activity and reducing MMP-9 expression (25). While another study indicated that curcumin inhibited TGF-β1 induced MMP-9 activity through Smad2, ERK1/2 and p38 pathways in the same cell type (26).

The interaction between the TME components is an important factor for BC prognosis, thus identifying the precise molecular mechanisms underlying metastasis is required. The biomarkers IL-6, IL-1β, TNF-α, TGF-β1, and MMP-9 demonstrated the strongest evidence of being associated with the overall trajectory patterns, as well as the severity, of lymphedema-fibrosis (27). TGF-β1 and/or TNF-α induce epithelial mesenchymal transition (EMT) in bronchial epithelial cells (28). Additive roles of TNF-α and TGF-β on the induction and activation of MMP-9 have been studied in human skin (29). Limited studies investigated the combined effects between TNF-α, TGF-β and MMP-9 in the TME. One study stated that breast cancer cell-secreted TNF-alpha and TGF-beta drive the expression of MMP-9 in stromal fibroblasts (30). Another study describes that enhancement of TAK1 activation by TGF-β1 and TNF-α synergistically induces epithelial to mesenchymal transition in breast cancer cells (31). This highlights the synergistic effect between TGF-β1 and TNF-α in promoting tumor formation and proliferation in non-invasive BC epithelial cells. Our study illuminates a novel axis of MMP-9 regulation mediated by the interaction of TGF-β and TNF-α. Utilizing the MDA-MB-231 breast cancer cell model, we demonstrate that the cooperative action of these cytokines significantly enhances MMP-9 expression through a previously unappreciated mechanism involving H3K36 dimethylation. This finding not only advances our understanding of MMP-9 regulation in breast cancer but also opens new avenues for therapeutic intervention targeting the epigenetic landscape of the TME.

By establishing the synergistic relationship between TGF-β and TNF-α in driving MMP-9 expression, our work sets the stage for the development of novel therapeutic strategies aimed at interrupting this pathway. The identification of H3K36me2 as a key epigenetic mark in this process further underscores the potential of targeting chromatin remodeling mechanisms in cancer therapy. As we continue to unravel the complexities of the TME, the insights gained from this study promise to pave the way for more effective and targeted approaches to combating breast cancer metastasis.

## 2 Materials and methods

Detailed material and methods are presented in the **Supplementary File**.

### 2.1 Immunohistochemistry

We purchased commercial breast cancer tissue microarray (TMA) from US Biomax Inc.(BRM961a) https://www.tissuearray.com/tissue-arrays/Breast/BRM961b; TissueArray.Com LLC 1(5885 Crabbs Branch Way, Derwood, MD 20855, USA. The tissue microarray (TMA) slide contained 48 cases of breast carcinoma, 36 metastatic carcinoma (35 matched with their primary breast carcinoma, 12 adjacent or adjacent normal breast tissue (**Supplementary Table S1**). Tissue arrays were commercially being sold by US Biomax Inc and were being used for various studies (https://www.tissuearray.com/Selected-References). Therefore, no ethical approval is needed. The tissue array Immunohistochemical staining was performed as described earlier (32, 33). Briefly, TMA slides were deparaffinized in xylene and rehydrated through descending grades of ethanol (100%, 95%, and 75%) to water. Antigen retrieval was performed by placing slides in target retrieval solution (pH6.0; Dako, Glostrup, Denmark) under a pressure cooker boiling for 8 min and cooling for 15 min. After washing in PBS, endogenous peroxidase activity was blocked with 3% H2O2 for 30 min and non-specific antibody binding was blocked with 5% nonfat milk for 1hr and 1% bovine serum albumin (BSA) solution for 1hr. Slides were treated overnight with primary antibodies (anti-TNF-α, 1:200, Cat# ab1793, Abcam, Cambridge, UK; anti-MMP-9, 1:200, Cat# ab38898, Abcam, Cambridge, UK; mouse anti-human TGF-β, 1:500, Cat# MCA 797, Bio-Rad, Hercules, California, USA) at room temperature using dilutions as recommended by manufacturers. After washing with PBS, slides were incubated for 1hr with a secondary antibody conjugated with HRP polymer chain (EnVision Kit, Dako, Glostrup, Denmark), and color was developed using 3,3′-diaminobenzidine chromogen substrate. Specimens were washed in running tap water, lightly counterstained with Harris hematoxylin, dehydrated through ascending grades of ethanol (75%, 95%, and 100%), cleared in xylene, and finally mounted in dibutyl phthalate xylene (DPX).

### 2.2 Slide scanning and scoring

The stained sample slides were evaluated under the light microscope by trained pathologist or researcher as described earlier (33). Semi-quantitative scoring systems are widely used to convert subject perception of IHC-marker expression into (semi)quantitative data, which is then used for statistical analyses and establishing of the conclusions. The existing clinical scoring process is based on two characteristics: overall staining intensity and the proportion of tissue or cells stained. The overall score of the staining intensity typically has four categories: negative (0), weak (1), moderate (2), and strong (3). H-score, Allred-score, and Immunoreactive score are considered as a “gold standard” of combined scoring system in IHC data evaluation and presentation. All these scoring systems use different categories for the proportion of tissues or cells stained (33). The H-score method takes into account both the area and intensity of staining to generate values between 0-300 using the following formula: Σ (1 × % cells staining weakly positive) + (2 × % cells staining moderately positive) + (3 × % cells staining strongly positive).

### 2.3 Quartile assignment based on TGF-β and TNF-α H-scores

For statistical comparisons, the human breast cancer tissue samples were categorized into 4 quartiles depending on TGF-β and TNF-α H-score values: first quartile (Low TNF-α- Low TGF-β), contained breast cancer tissues, which have H-score values: TNF-α <169.7- and TGF-β <223.2; second quartile (Low TNF-α- High TGF-β), contained breast cancer tissues, which have H-score values: TNF-α: <169.7 and TGF-β: ≥223.2; third quartile (High TNF-α- Low TGF-β), contained breast cancer tissues, which have H-score values: TNF-α: ≥169.7 and TGF-β: <223.2; fourth quartile (High TNF-α- High TGF-β), contained breast cancer tissues, which have H-score values: TNF-α: ≥169.7 and TGF-β: ≥223.2. All quartiles are shown in the form of scatter plot graphs for TGF- β and TNF- α (in the result section 3.2).

### 2.4 Cell culture

The MDA-MB-231 is a well-established cell line model for triple-negative breast cancer. It is commonly used to identify genes and pathways that are linked to specific metastatic sites. Human MDA-MB-231 cells were purchased from American Type Culture Collection (ATCC. Manassas, VA, USA), and then grown using standard protocol. Cell stimulation was done as described in the detailed methodology section in the **Supplementary File**.

### 2.5 Real-time quantitative PCR

RNA isolation from MDA-MB-231 cells, cDNA synthesis, real-time PCR and protein analysis were performed using standard protocols. Primer details are described in the **Supplementary File**.

### 2.6 MMP-9 determination

Secreted MMP-9 protein in supernatants of MDA-MB-231 cells stimulated with vehicle, TGF-β, TNF-α or TGF-β/TNF-α was quantified using Human MMP-9 Quantikine ELISA Kit (DMP900, R&D Systems, Minneapolis, MN, USA) following the manufacturer’s instructions (34).

### 2.7 Western blotting

MDA-MB-231 cells were treated with TGF-β/TNF-α and were harvested after incubation. Western blotting was performed on cell lysates using standard protocol. Detailed procedure along with antibodies’ information was described in material methods section in **Supplementary File**.

### 2.8 Small interfering RNA transfection

Transient transfection of MDA-MB-231 cells was done using Lipofectamine RNAiMAX reagent (Themo Fischer, USA) following the manufacturer’s instructions and was transfected separately with Smad2-siRNA (20 nM; s8397, Thermo Fischer, USA), Smad3-siRNA (20 nM; s8401, Thermo Fischer, USA), p38-siRNA (20 nM; s3538, Thermo Fischer, USA), NSD2 (20 nM; s526860, Thermo Fischer, USA) and scramble (negative control) siRNA (20 nM; 4390843, Thermo Fischer, USA). After 48 hours of transfection, cells were treated with vehicle, TGF-β, TNF-α or TGF-β/TNF-α and incubated for 24 hours. Cells and conditioned medium were harvested for RNA isolation and ELISA. The knockdown of Smad 2/3, nuclear receptor binding SET domain protein 2 (NSD2), and p38 pathways was assessed using Real-Time PCR gene-specific primer probes.

### 2.9 Zymography

MDA-MB-231 cells were incubated with TGF-β/TNF-α. After incubation for 24 hours, conditioned media were collected and mixed with zymogram sample buffer. Zymography was performed using standard protocol.

### 2.10 Histone modification multiplex assay

MDA-MB-231 cells were treated with TGF-β, TNF-α or TGF-β/TNF-α and were harvested after 6 h incubation. Total Histone extracts were prepared using the EpiQuick Total Histone Extraction kit (EpigenTek, Farmingdale, NY, USA) as per the manufacturer’s instructions. The concentration of the histone extracts was measured by QuickStart Bradford Dye Reagent, 1x Protein Assay Kit (Bio-Rad Laboratories, Inc, CA). Histone H3 modifications triggered by TGF-β/TNF-α treatment were then screened in 100 ng of total histone extract per well of the assay plate using Histone H3 Modification Multiplex Assay Kit (Abcam, Cambridge, UK) following the manufacturer’s instructions.

### 2.11 Chromatin immunoprecipitation

Chromatin immunoprecipitation (ChIP) was performed using the SimpleChIP enzymatic ChIP kit (catalog no. 9003, CST) following the manufacturer’s instructions. Briefly, MDA-MB-231 cells stimulated with TGF-β and TNF-α were crosslinked with formaldehyde and digested with micrococcal nuclease followed by sonication to yield fragments ranging from 200 to 800 bp using a Covaries system. The digested chromatin fragments were subjected to immunoprecipitation using primary Abs specific to H3K36Me2 (catalog no. ab9049, Abcam), histone H3 (positive IP control, catalog no. 4620, CST), and normal rabbit IgG (negative IP control, catalog no. 2729, CST), for overnight at 4°C and incubated with protein G magnetic beads for 2 h at 4°C. We eluted the chromatin from an Ab/protein G magnetic beads complex by incubation at 65°C for 30 min and by magnetic separation. We then reverse crosslinked the chromatin by treating with Proteinase K for 2 h at 65°C and purified DNA from the ChIP fraction using the spin column method. The enrichment of DNA sequences was then detected by real-time quantitative PCR (qPCR) using SYBR Green mix and EpiTect ChIP-qPCR primers (GPH1008476(-)05A, GPH1008476(-)04A, GPH1008476(-)01A, Qiagen) spanning the MMP-9 gene promoter region.

### 2.12 Statistical analysis

Statistical analyses were performed using GraphPad Prism software (version 6.07, La Jolla, CA, USA). Data are shown as ± standard error of the mean (SEM), unless otherwise indicated. Student T Test and One-way ANOVA followed by Tukey’s test were used to compare means between groups. For all analyses, data from a minimum of three sample sets were used for statistical calculation, p-value <0.05 was considered significant. No significance (ns), (35).

## 3 Results

### 3.1 TGF-β potentiates TNF-α-induced MMP-9 expression in breast cancer MDA-MB-231 cells

**Figure 1:**
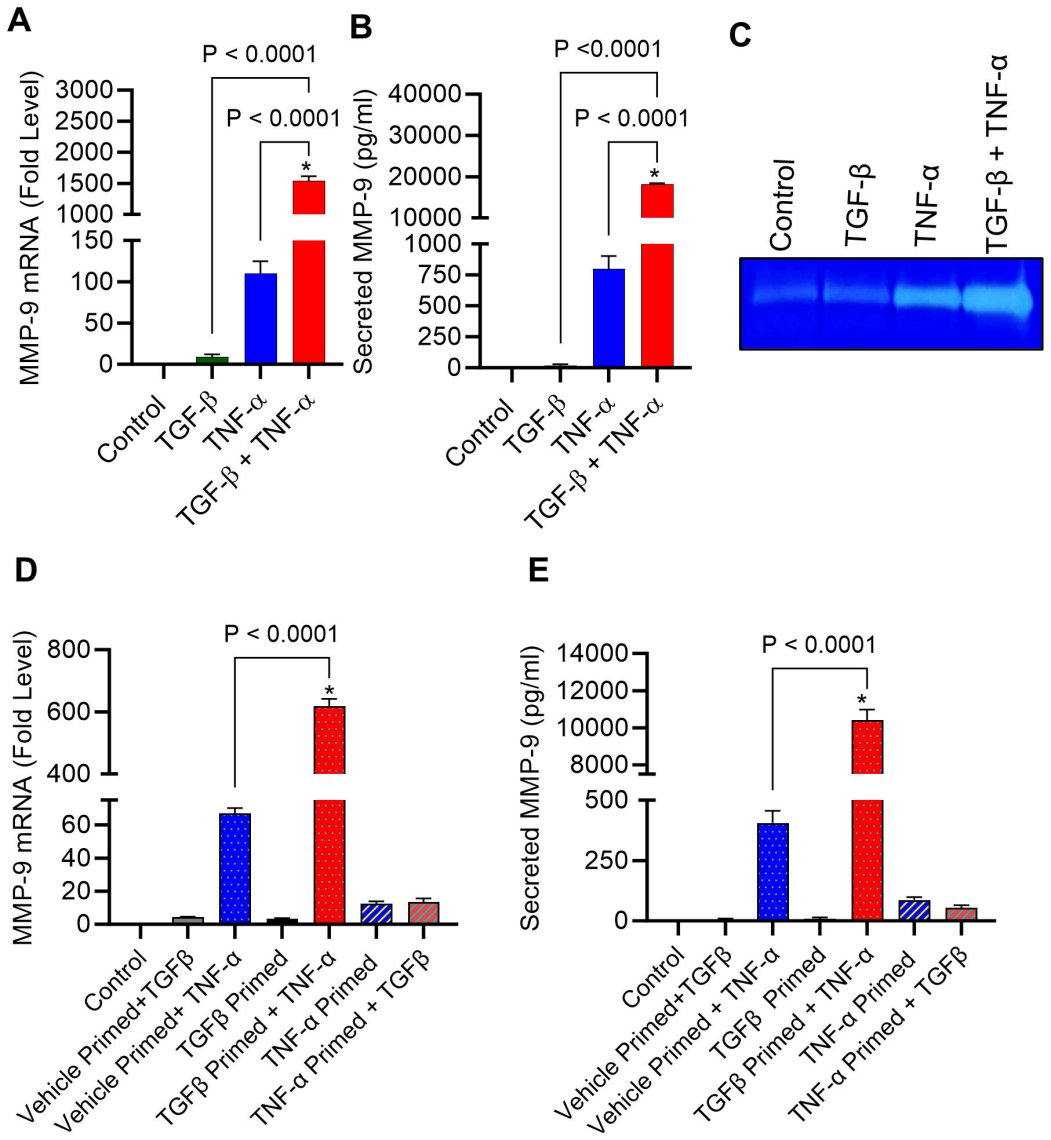
TGF-β boosts TNF-α-induced MMP-9 expression in human MDA-MB-231 cells. MDA-MB-231 cells were cultured in 12-well plates with a cell concentration set at 0.3×10^6^ cells per well. Cells were subjected to a 24 h treatment with vehicle, TGF-β (20 ng/mL), TNF-α (10 ng/mL), or TGF-β/TNF-α. Cells and supernatants were collected following incubation period. **(A)** The isolation of total RNA was followed by the assessment of MMP-9 mRNA expression using real-time PCR. **(B)** The concentration of secreted MMP-9 protein in the culture media was assessed by ELISA. **(C)** Gelatin zymography was conducted on conditioned media to examine the activity of MMP-9. **(D, E)** After a 5 h priming with TGF-β or TNF-α, cells were washed with media and subsequently treated with TNFα or TGFβ for 24 h, respectively. MMP-9 expression was determined. All data is presented as the mean ± SEM (n= 3). *p < 0.05.

MMP-9 expression is elevated in TME and is directly correlated with clinical outcome of tumor invasion and metastasis (11). It remains unclear how MMP-9 expression is upregulated in TME. Since both TGF-β and TNF-α are drivers of inflammation and cancer metastasis, and are co-expressed with MMP-9, we asked whether the co-exposure of breast cancer cells to TGF-β and TNF-α could amplify MMP-9 expression in these cells. To assess this, MDA-MB-231 breast cancer cells were used as a representative model as this cell line has been widely used in breast cancer research previously (36). The cells were stimulated for 24h with TGF-β and TNF-α, alone or in combination, and MMP-9 expression was assessed at the transcriptional and translational levels. The data show that compared with control or TGF-β alone stimulation, TNF-α alone stimulation induced a significant upregulation in MMP-9 mRNA (**Figure 1A**) and secreted protein (**Figure 1B**); however, the co-stimulation with TGF-β and TNF-α synergistically promoted MMP-9 gene/protein expression (**Figures 1A, B**). As expected, zymography revealed substantially increased gelatinolytic activity (concordant with high MMP-9 expression) in the conditioned media from the cells co-stimulated with TGF-β and TNF-α, compared with TNF-α alone stimulation (**Figure 1C**). Next, we asked whether TGF-β/TNF-α combined treatment of MDA-MB-231 breast cancer cells synergized the transcription of other MMPs. To test our hypothesis, we measured the gene expression of MMP-2 and MMP-25. Our results show that TGF-β/TNF-α combined treatment of the cancer cells synergistically increased the expression of MMP-2 and MMP-25 when we compared either treatment of TGF-β or TNF-α (**Supplementary Figures S1A, B**). To further analyze the priming effect of TGF-β on TNF-α stimulation, cells were first treated with TGF-β for 5h, and then TNF-α was added for an additional 24h. It is evident that TGF-β priming amplified the TNF-α-induced MMP-9 expression. However, a similar priming with TNF-α (5h), followed by TGF-β stimulation did not increase MMP-9 expression (**Figures 1D, E**). To test the generalizability of our findings across different breast cancer cell lines, we treated breast cancer cell lines MCF-7 and T47D with TGF-β or TNF or TGF-β/TNF-α. We found that combined treatment of TGF-β/TNF-α synergistically upregulates the production of MMP-9 by the MCF-7 breast cancer cell line (**Supplementary Figure S2A**). However, these observations were not seen in T47D cells. MMP-9 was not detected in the culture supernatant of T47D cells (**Supplementary Figure S2B**). Altogether, these results demonstrate that TGF-β priming amplifies the TNF-α-mediated induction of MMP-9 in breast cancer cells.

### 3.2 MMP-9 expression associates with TNF-α and TGF-β expression in the human breast tissues

**Figure 2:**
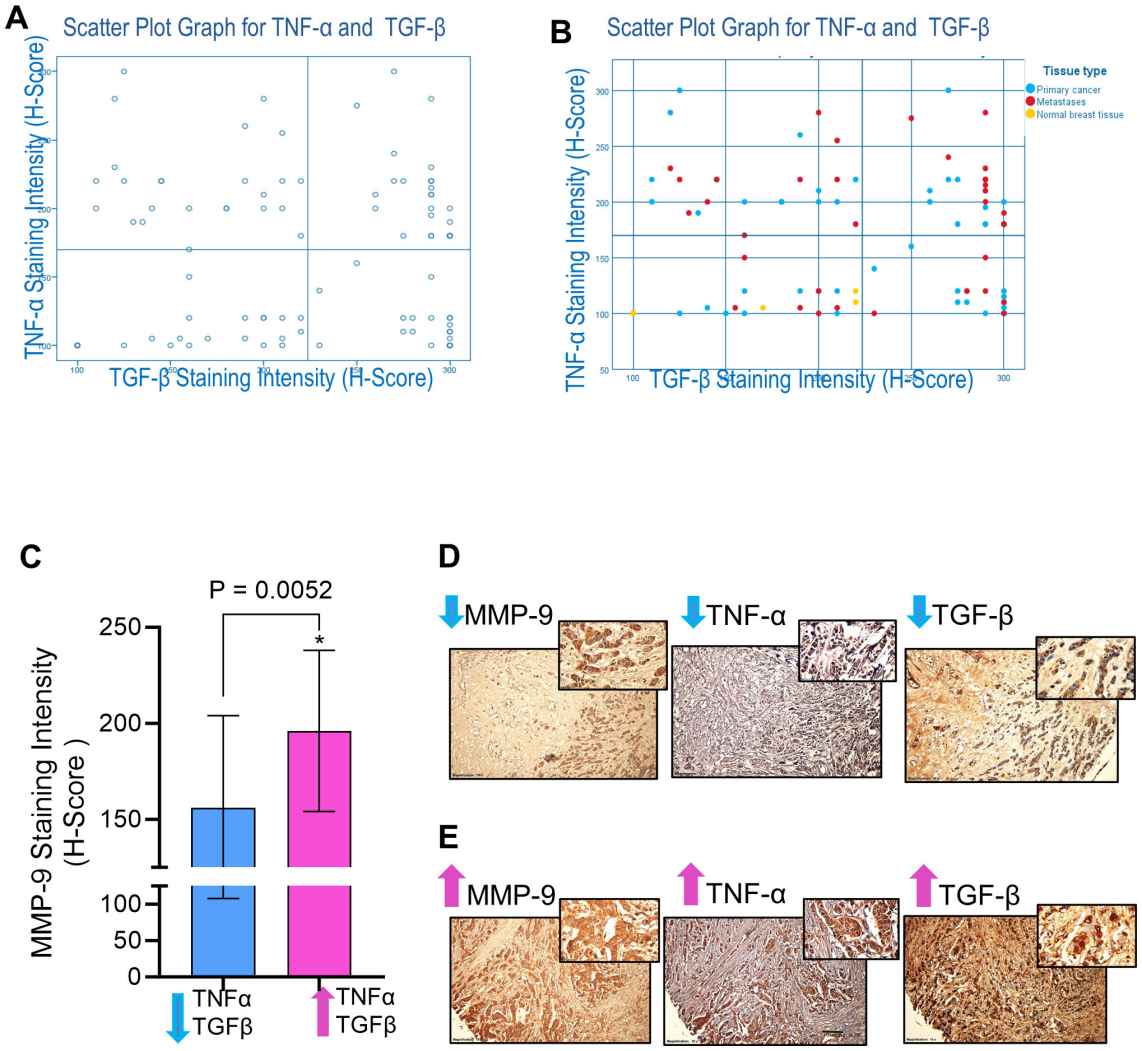
MMP-9 expression is associated with TGF-β and TNF-α in breast cancer tissues. Immunohistochemical staining was done for MMP-9, TGF-β, and TNF-α expressions. Tissue microarray TMA slides (88 breast cancer tissues from breast cancer patients) were subjected to immunohistochemistry using antibodies against MMP-9, TGF-β, and TNF-α. Based on IHC staining intensities of TGF-β and TNF-α, patients’ samples were divided into four groups (Low TNF-α- Low TGF-β; Low TNF-α- High TGF-β; High TNF-α- Low TGF-β; High TNF-α- High TGF-β. **(A)** Scatter Plot Graph for TNF-α and TGF-β. **(B)** Scatter Plot Graph for TNF-α and TGF-β with indication of patients with primary cancer, metastasis and normal breast cancer tissues. **(C)** MMP-9 expression was elevated in the breast cancer tissues (n=24 patients) having high levels of TGF-β and TNF-α compared to the tissues (n=20 patients) having less TGF-β and TNF-α. **(D)** Representative IHC images of the samples of breast cancer tissues with low levels of MMP-9, TGF-β and TNF-α. **(E)** Representative IHC images of the samples of breast cancer tissues showing high levels of MMP-9, TGF-β and TNF-α. All data is presented as the mean ± SD. *p < 0.05.

MMP-9, TNF-α, and TGF-β are integral contributors to the complicated and multifaceted process of breast cancer metastasis (17, 37). Since MMP-9 overexpression facilitates the spread of cancer, understanding the intricate interplay between MMP-9 and other coplayers in breast cancer metastasis, such as TNF-α and TGF-β, is imperative to developing targeted therapeutic strategies to intercept the pro-metastatic pathways and alleviate breast cancer metastasis. Our *in vitro* data show that TNF-α and TGF-β cooperatively upregulate MMP-9 expression in breast cancer cells. Next, we asked whether this relationship was reflected in clinical analysis of human cancer tissue samples. To test whether MMP-9 overexpression in breast cancer tissues was associated with TNF-α and TGF-β expression, IHC analysis was done on 48 women breast cancer tissue samples. Based on IHC staining intensities of TGF-β and TNF-α, patients’ samples were divided into four groups (Low TNF-α- Low TGF-β; Low TNF-α- High TGF-β; High TNF-α- Low TGF-β; High TNF-α- High TGF-β: **Figures 2A, B**). The one-way analysis of variance (ANOVA) of MMP-9 expression reveals that there is a significant difference between the groups (F= 5.21; p =0.002). Breast cancer tissue samples that stained intensely for TNF-α and TGF-β expression revealed markedly increased MMP-9 expression (**Figure 2C**). Representative IHC images are shown in **Figures 2D, E**. Concordantly, the samples that displayed low expression of TNF-α and TGF-β exhibited low expression of MMP-9. Taken together, co-expression of elevated MMP-9 with of TGF-β and TNF-α in the human breast cancer tissues is corroborated to our *in vitro* findings indicating the synergistic role of TGF-β and TNF-α in potentiating MMP-9 in breast cancer cells.

### 3.3 TGF-β augments TNF-α induced MMP-9 expression through the SMAD2/3 and p38 pathways

**Figure 3:**
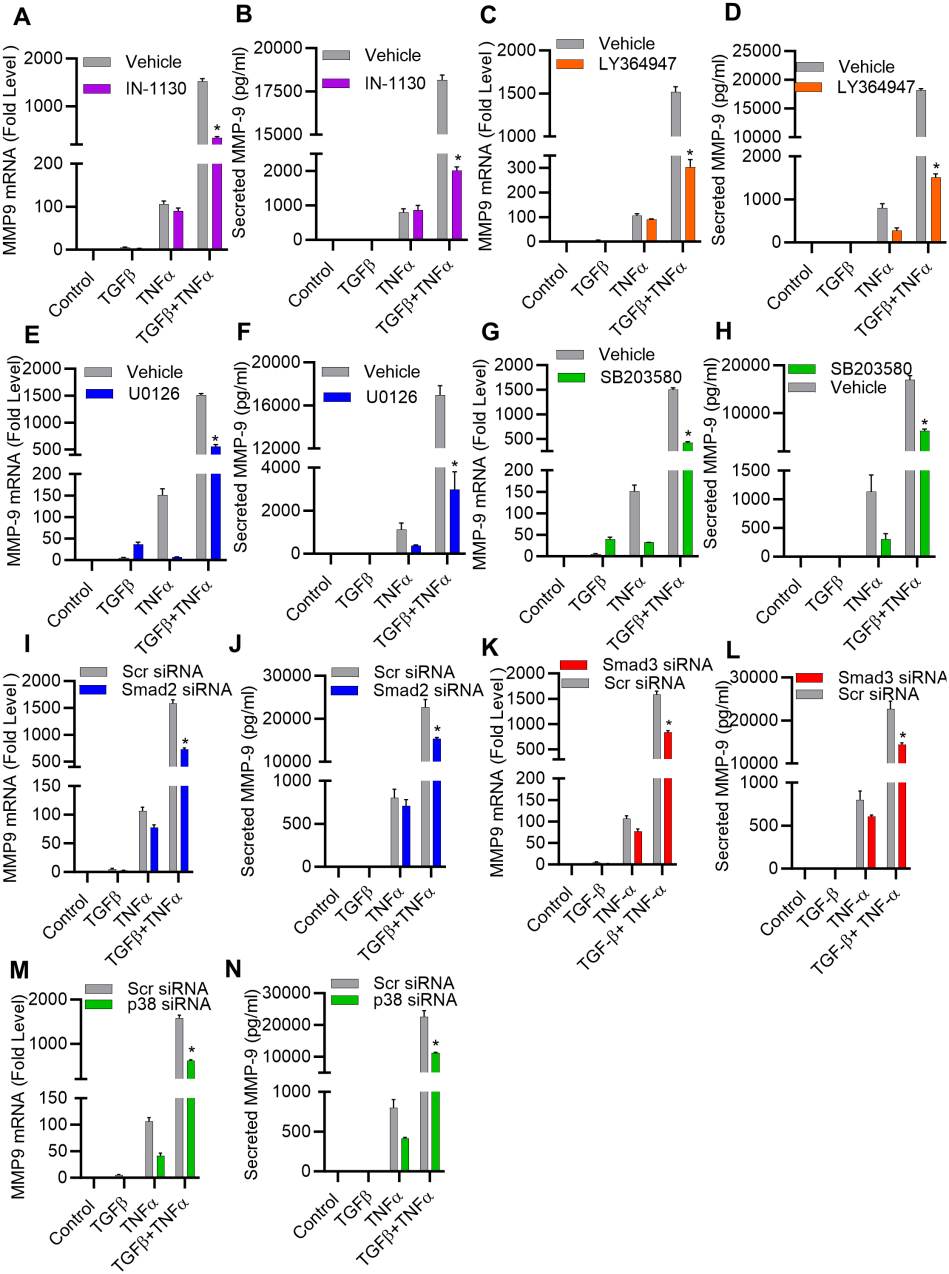
TGF-β potentiates the expression of MMP-9 induced by TNF-α, involving both the SMAD2/3 and p38 pathways. **(A–H)** MDA-MB-231 breast cancer cells were pretreated for 1 h with SMAD2/3 (IN-1130; 10 µM), TGFβR1 (LY364947; 10 µM), MEK1/2 (U0126; 10 µM), p38 (SB203580; 10 µM) inhibitors and then incubated with TGF-β, TNFα or TGF-β/TNF-α for 24 h. Both cells and their culture media were collected. MMP9 mRNA and protein expression were determined. **(I–N)** Knockdown of SMAD 2/3 and P38 pathway attenuate the potentiating effect of TGFβ. Knocking down SMAD 2, SMAD 3 or p38 results in a decrease in TGFβ/TNF-α induced MMP9 expression. Transfection of MDA-MB-231 cells was carried out using SMAD2 siRNA (20 nM), SMAD3 siRNA (20 nM), p38 siRNA (20 nM), or scrambled siRNA (20 nM) following the procedures detailed in the Materials and Methods section. Measurement of SMAD 2, SMAD 3 or p38 mRNA was carried out through real-time RT-PCR after a 36-hour interval. Cells with knocked-down SMAD 2, SMAD 3, or p38 were subjected to incubation with TGFβ, TNF-α, or TGFβ/TNF-α. MMP9 mRNA and protein expression were determined. All data is presented as the mean ± SEM (n= 3). *p < 0.05.

Given the predominant role of TGF-β priming in this synergy, we next analyzed the potential involvement of TGF-β signaling pathways in TNF-α-mediated induction of MMP-9 in MDA-MB-231 cell model. Notably, cellular responses to TGF-β are orchestrated through Smad and MAPK signaling pathways (38). To test the role of Smad in this potentiating effect, MDA-MB-231 cells were treated with inhibitors of Smad2/3 (IN-1130) and TGFβR1 (LY364947) before treatments with TGF-β, TNF-α, or TGF-β/TNF-α and the data show that Smad2/3 or TGF-βR1 inhibition significantly mitigated the potentiating effect of TGF-β on TNF-α-mediated MMP-9 expression (**Figures 3A–D**). Moreover, this potentiating effect of TGF-β was also significantly attenuated by pre-treating cells with inhibitors of MEK1/2 (U0126) and p38 MAPK (SB203580) (**Figure 3E–H**). In contrast, inhibition of the JNK signaling pathways had no significant effect (**Supplementary Figures S3A, B**). Together, these findings suggest that both ERK1/2 and p38 MAPK pathways co-operate with the Smad signaling pathway in amplifying TNF-α-induced MMP-9 expression. To further confirm the obligatory requirement of Smad signaling for TGF-β potentiating effect on TNF-α-induced MMP-9 expression, cells were individually transfected with specific siRNAs targeting genetic suppression of Smad2 or Smad3, and compared to scrambled siRNA control. There was approximately a 50% decrease in the Smad2/3 mRNA levels was achieved with siRNA (**Supplementary Figures S4A, B**). Expectedly, Smad2/3 genetic suppression also downmodulated the potentiating effect of TGF-β on TNF-α-induced MMP-9 expression in MDA-MB-231 cells (**Figures 3I–L**). Next, the effect of the genetic suppression of p38 MAPK on synergistic induction of MMP-9 by TGF-β and TNF-α co-stimulation was also assessed. In this regard, the cells transfected with p38 siRNA exhibited a significant reduction in p38 mRNA expression, compared to expression in scrambled siRNA-transfected control (**Supplementary Figure S4C**). Validating the outcome of p38 inhibition, the p38 deficiency also led to a significantly reduced expression of MMP-9 mRNA and protein, following co-stimulation with TGF-β and TNF-α (**Figures 3M, N**).

**Figure 4:**
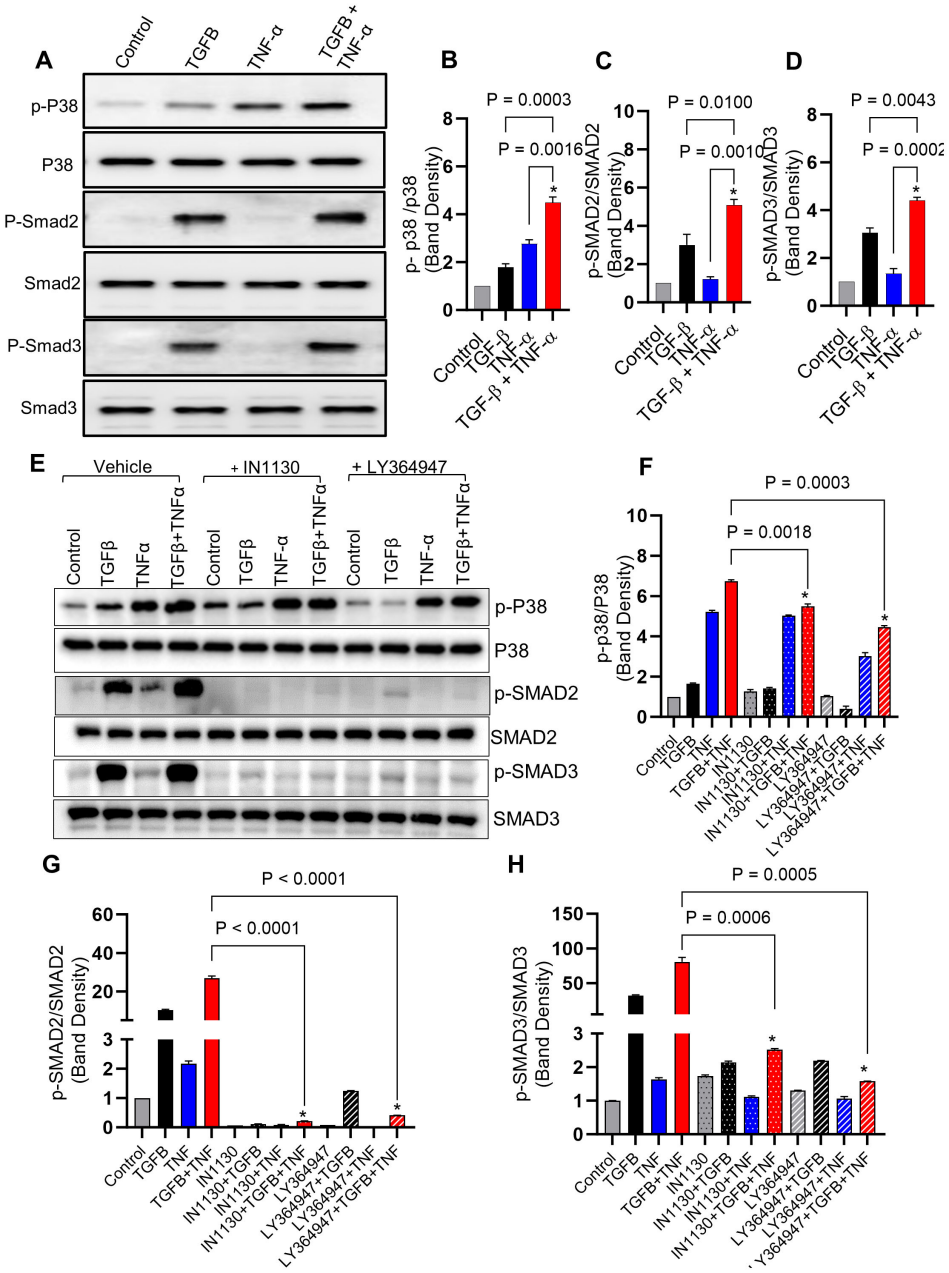
Combined treatment of TGF-β/TNF-α enhances p38 and Smad2/3 phosphorylation. **(A)** Cells were treated with vehicle, TGF-β, TNF-α or TGF-β/TNF-α and cell lysates were prepared and run on denaturing gel as described in the materials and methods section. Phosphorylated p38 and Smad2/3 are presented in the upper panels, while the lower panels exhibit the total respective proteins. **(B–D)** Calculations of the ratio between phosphorylated and total protein ratios were based on densitometry data, relative to the control. **(E)** Inhibitors blocking the TGF-β pathway lead to a reduction in p38 and SMAD2/3 phosphorylation induced by TGF-β/TNF-α. MDA-MB-231 cells were pretreated with SMAD2/3 (IN-1130; 10µM) and TGFβR (LY364947; 10µM) inhibitors for 1 h before being incubated with TGF-β, TNF-α, or TGF-β/TNF-α. The upper panels display phosphorylated p38 and SMAD2/3, while the lower panels exhibit the total corresponding proteins. **(F–H)** Densitometry data were used to calculate the ration between the phosphorylated and total protein ratios relative to the control. All data is presented as the mean ± SEM (n= 3). *p < 0.05.

Next, we determined activation of the p38 and Smad2/3 signaling pathways in response to TGF-β/TNF-α co-stimulation in MDA-MB-231 breast cancer cells. Our results show that the combined stimulation with TGF-β and TNF-α potentially enhanced phosphorylation of p38 and Smad2/3 (**Figures 4A–D**), as compared to individual stimulations with TGF-β or TNF-α. Regarding phosphorylation changes in response to Smad2/3 or TGFβR pathway blocking using specific inhibitors (IN-1130 and LY364947, respectively), we found that intercepting the Smad2/3- or TGFβR-associated signaling significantly reduced the phosphorylation levels of Smad2/3 and p38 (**Figures 4E–H**), confirming the critical role of p38 and Smad2/3 signaling in the induction of MMP9 by TGF-β/TNF-α co-stimulation. Taken together, these genetic and pharmacologic lines of evidence support that both Smad2/3 and p38 signaling molecules participate in the synergistic effect of TGF-β/TNF-α co-stimulation on MMP-9 production.

### 3.4 Epigenetic modifications triggered by TGF-β/TNF-α co-stimulation

**Figure 5:**
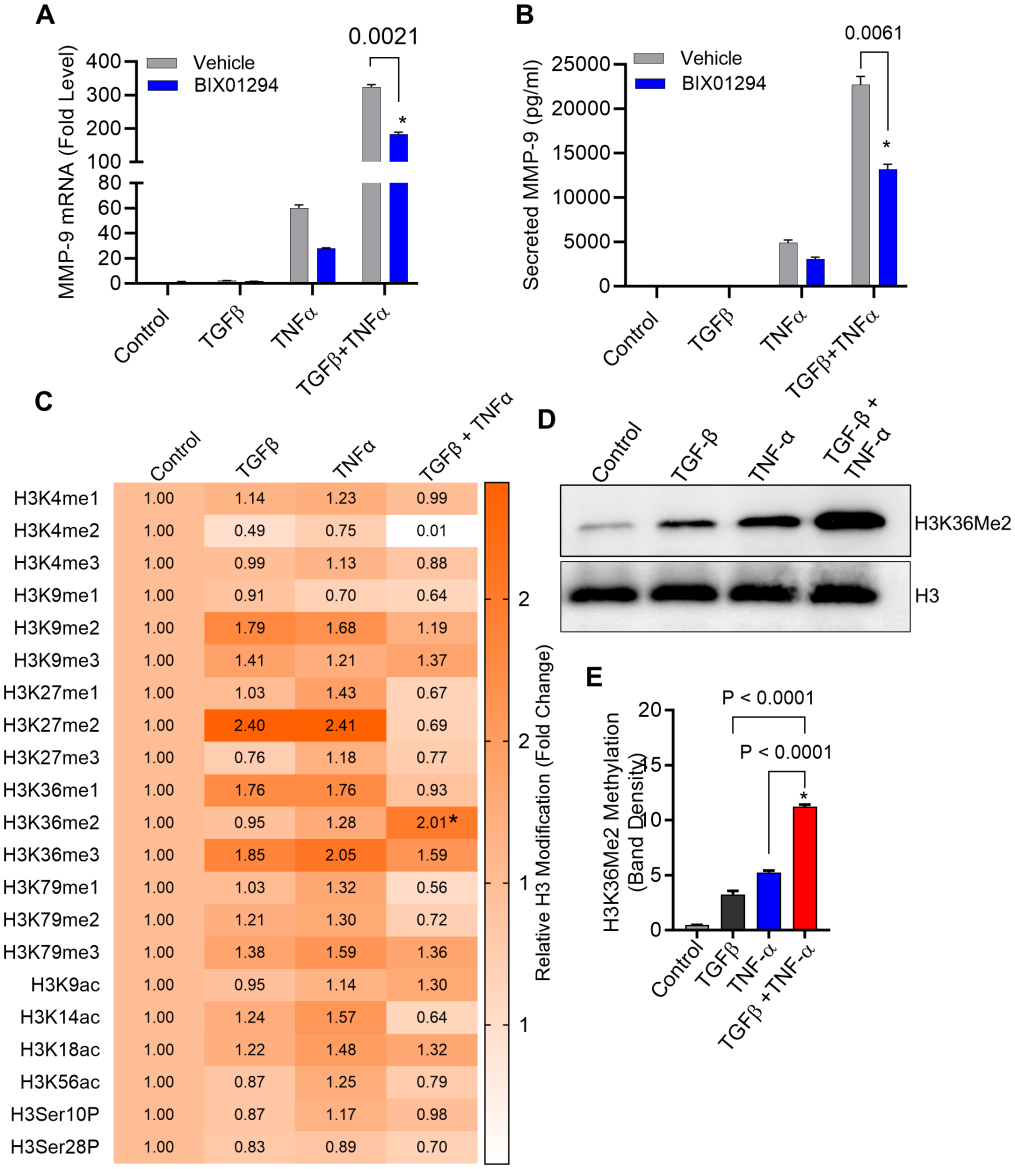
Histone methylation is increased in breast cancer cells following treatment with TGF-β/TNF-α. **(A, B)** Cells were treated with BIX 01294 (10µM) for 1 h and then treated with TGF-β/TNF-α for 24 h. Cells and culture supernatant were collected. MMP9 mRNA was quantified by RT-PCR. Secreted MMP9 protein in culture media was determined by ELISA. **(C)** Cells were subjected to treatment with either vehicle, TGFβ, TNFα, or TGFβ/TNFα for a period of 3 hours. Isolated histone extracts underwent assessment for histone modification levels at 21 diverse sites through the EpiQuick Histone H3 modification colorimetric assay kit, and the outcomes are displayed as a Heatmap. **(D)** After a 3-hour exposure to vehicle, TNF-α, or TGF-β, MDA MB 231 cells were analyzed for histone methylation levels using Western blot. **(E)** Quantification of Western blots. Data are presented as mean ± SEM values (n=3). *p < 0.05.

Epigenetic alterations, such as histone acetylation and methylation at lysine residues, play a key role in regulating gene expression levels. Since TGF-β signaling pathway is linked with epigenetic alterations (39), we asked whether the potentiating effect of TGF-β on TNF-α-mediated induction of MMP-9 expression in MDA-MB-231 cells involved peculiar epigenetic alterations. To investigate the impact of epigenetic reprogramming on this event, we used inhibitors for histone acetylation, histone methylation and DNA methylation. Our results show that inhibition of histone methylation by BIX 01294 attenuated the synergistic effect of TGF-β/TNF-α co-stimulation on MMP-9 expression in MDA-MB-231 breast cancer cells (**Figures 5A, B**). Similar results have been seen in MCF-7 breast cancer cells when treated with BIX 01294 before TGF-β/TNF-α co-stimulation (**Supplementary Figure S5A**). However, blocking of histone acetylation did not change the effect of TGF-β alone stimulation on MMP-9 expression (**Supplementary Figures S6A–D**). Next, to identify specific histone methylations sites, a comprehensive profiling of histone modifications was performed. To achieve this, we utilized the EpiQuik™ Histone H3 Modification Multiplex Assay Kit, allowing us to examine 21 of the most well-established patterns of H3 modification. To this end, histone dimethylation (me2) at H3K36 was found to be higher in cells stimulated with TNF-α than in control cells. Importantly, the co-stimulation with TGF-β and TNF-α led to a significantly increased histone dimethylation at this site, compared to TNF-α stimulation alone (**Figure 5C**). For validation, we further confirmed this H3K36Me2 hypermethylation using Western blotting and found that TGF-β/TNF-α co-stimulation induced a significantly higher dimethylation at H3K36, compared to control as well as against individual stimulations with TGF-β or TNF-α (**Figures 5D, E**). However, such results were not seen in MCF-7 breast cancer cells (**Supplementary Figure S5B**). Overall, the data from both pharmacological inhibition of histone methylation and comprehensive profiling of histone modifications support that the H3K36Me2 hypermethylation, driven by synergy between TGF-β and TNF-α, is implicated in MMP-9 overexpression in late-stage breast cancer MDA-MB-231 cell model.

### 3.5 NSD2 histone methyltransferase knockdown effectively inhibits MMP-9 expression co-induced by TGF-β and TNF-α

**Figure 6:**
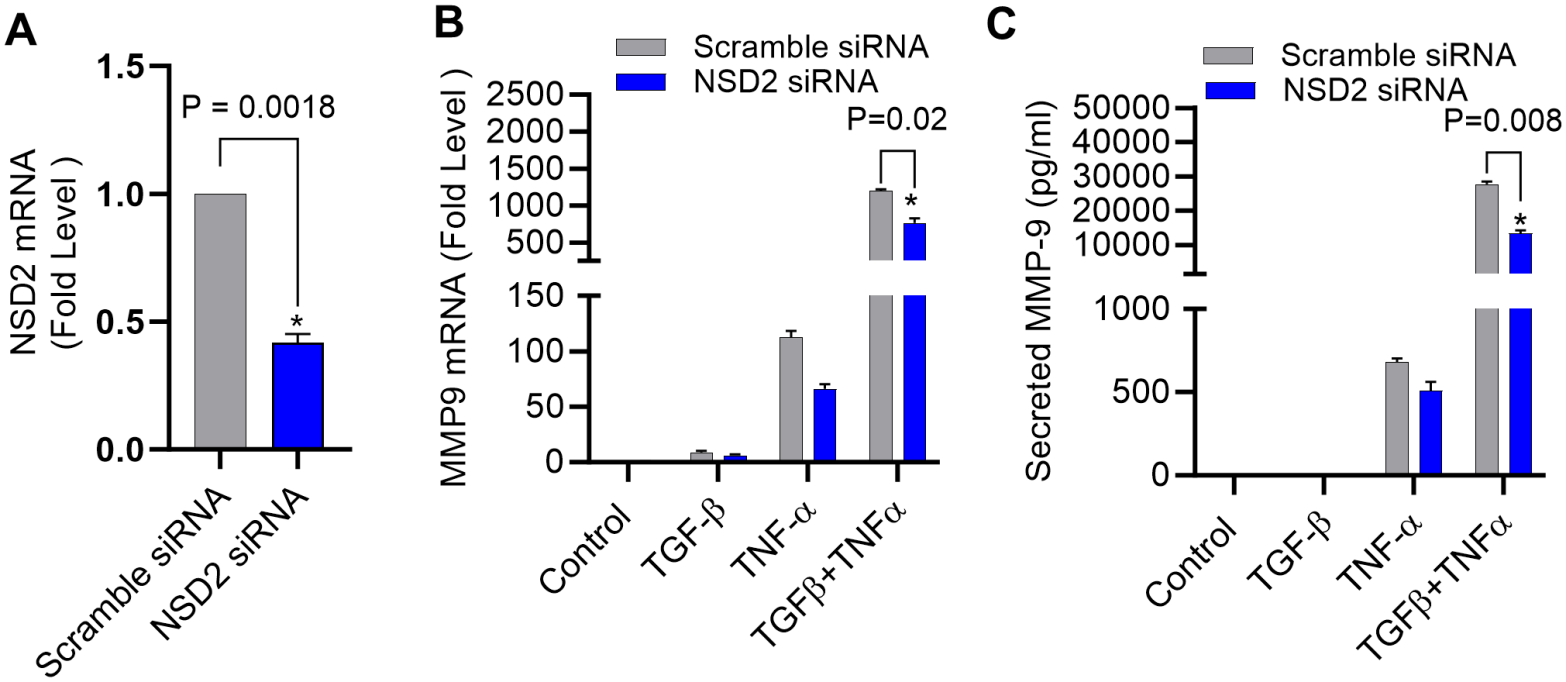
Knockdown of NSD2 histone methyltransferase suppresses TGF-β/TNF-α induced MMP-9 expression. MDA-MB-231 cells were transfected with either NSD2 siRNA (20 nM) or scrambled siRNA (20 nM) as described in the Materials and Methods section. **(A)** After 36 hours, real-time RT-PCR was performed to measure NSD2 mRNA. **(B, C)** MDA-MB-231 cells deficient with NSD2 were incubated with vehicle, TGF-β, TNF-α or TGF-β/TNF-α. Cells and supernatants were collected and MMP9 mRNA and protein were measured by real-time RT-PCR and ELISA respectively. All data are presented as mean ± SEM (n=3). *p < 0.05.

NSD2 histone methyltransferase dimethylates the DNA packaging protein histone H3 at the 36^th^ lysine residue (H3K36me2) (40) and elevated NSD2 protein expression has been observed in various human cancers, often linked to tumorigenesis (41, 42). To validate that the synergistic expression of MMP-9 in our cell model was largely dependent on histone methylation, NSD2 was genetically suppressed using specific siRNA transfections, resulting in ∼60% reduction in NSD2 mRNA levels, compared to control (scramble siRNA) (**Figure 6A**). Of note, NSD2 deficiency in the cells led to a significant reduction in MMP-9 gene/protein expression in TGF-β/TNF-α co-stimulated cells (**Figures 6B, C**). Next, we also identified that the TGF-β/TNF-α mediated synergistic transcription of other MMPs (MMP-2, MMP-25) were also affected by suppressing H3K36 dimethylation through NSD2 siRNA transfection (**Supplementary Figure S1A, B**). Taken together, these findings underscore the predominant role of NSD2 histone methyltransferase in H3K36 dimethylation, following TGF-β/TNF-α co-stimulation in MDA-MB-231 cells.

### 3.6 TGF-β/TNF-α co-stimulation promotes the H3K36 dimethylation at MMP-9 promotor

**Figure 7:**
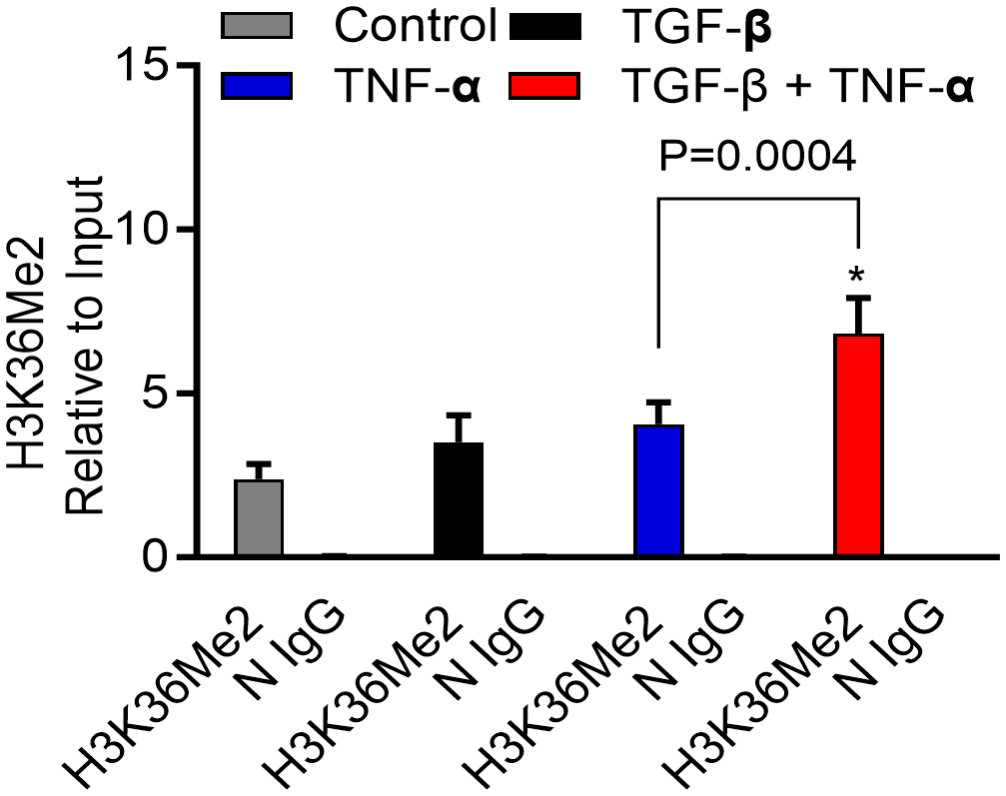
H3K36Me2 methylation at the MMP-9 promoter site is elevated by TGF-β/TNF-α. Determination of histone modifications at the MMP9 promoter was carried out by analyzing chromatin that underwent immunoprecipitation with antibodies H3K36Me2 or IgG (as a control) Ab. Levels of histone modifications were measured using PCR primer specific to the MMP-9 promoter region. Differences are presented as fold enrichment levels. Data are expressed as mean ± SEM (n=3). *p < 0.05.

We found that TGF-β/TNF-α co-stimulation resulted in hypermethylation at histone H3K36 residues in breast cancer cells, compared to cells treated with TGF-β or TNF-α alone. These results reflect the status of overall histone methylation in the cells. Next, we asked if the similar H3K36Me2 hypermethylation was detected at the MMP-9 promoter region, following TGF-β/TNF-α co-stimulation. To address this, ChIP-qPCR was used and the specificity of each ChIP was validated using an IgG control. As expected, ChIP-qPCR analysis showed significantly increased H3K36Me2 methylation at the MMP-9 promoter region in cells that were co-stimulated with TGF-β and TNF-α, compared to cells stimulated with TGF-β or TNF-α alone (**Figure 7**). Altogether, these results suggest that the H3K36 dimethylation plays a crucial role in *MMP-9* gene regulation in breast cancer cells, induced by TGF-β/TNF-α co-stimulation.

## 4 Discussion

The compelling association of MMP-9 with tumor invasiveness and poor prognostic outcomes has long underscored its significance in breast cancer (11, 43, 44). However, the intricate regulatory mechanisms dictating MMP-9 expression within the TME have remained elusive until now. Although it is known that TGF-β or TNF-α stimulation regulates the MMP-9 expression in various cells, it remains unclear how the combined TGF-β/TNF-α stimulation modulates MMP-9 expression in breast cancer cells. Individually, MMP-9, TNF-α, and TGF-β are integral contributors in the complicated and multifaceted process of breast cancer metastasis (17, 45, 46). In light of the identified elevated levels of TGF-β and TNF-α in the TME, we sought to examine whether these cytokines cooperatively promoted MMP-9 expression. Our findings reveal that the interaction between TGF-β and TNF-α significantly amplifies MMP-9 expression in human MDA-MB-231 breast cancer cells, underscoring a complex regulatory network that extends beyond individual cytokine actions to their cooperative impact. These findings were corroborated by the breast cancer tissue samples, which demonstrated elevated levels of MMP-9 expression, associated with high expression of TGF-β and TNF-α. In line with this observation, Han et al. reported that TNF-α and TGF-β synergistically induced pro-MMP-9 expression in human skin as well as in isolated dermal fibroblasts and epidermal keratinocytes (47). Also, MMP-9 expression in the tumor fibroblasts was found to be directly induced by tumor cell-derived TNF-α, EGF, or TGF-β (30). However, none of these studies addressed the mechanistic events that orchestrated the synergy between TGF-β and TNF-α for MMP-9 upregulation. Using an MDA-MB-231 cell line model of breast cancer, we herein delve into the epigenetic mechanisms that drive the synergy between TGF-β and TNF-α, with implications for MMP-9 overexpression in the TME.

Having found that TGF-β priming was predominantly involved in potentiating the effect of TNF-α stimulation in MMP-9 expression, we first evaluated the role of TGF-β signaling pathways in this synergy. TGF‐β binds to its cognate receptor complex comprising of type I (TGF‐βRI; ALK5) and type II receptor (TGF‐βRII). TGF‐βRI and TGF‐βRII function in concert in an interdependent manner. Our data show that TGF‐βRI inhibition is sufficient to limit the synergy between TGF-β and TNF-α, leading to a significant suppression of MMP-9 expression in the cells. Our results bear some similarities with those of a previous study showing that TGF-β1/TNF-α co-induced epithelial to mesenchymal transition (EMT) and invasiveness of breast cancer cells, through gradually increasing TGF-β receptors expression (31). The cognate receptor-ligand stimulation by TGF‐β activates the downstream signaling events by phosphorylating cytoplasmic mediators Smad2/3, which form a complex with Smad4 and translocate to the nucleus and regulate transcriptions of target genes (48). Our data show that TGF-β-mediated phosphorylation of Smad2/3 was increased by TNF-α. Dong et al., reported that increased expressions of Smad4, Smad2/3, and phosphorylation of Smad2 were noticed in MMP-9 expressor cell lines (24). Certain similarities to observations from this study are evident from our results. Our data clearly show that blocking the binding of TGF‐β to its cognate receptor by specific inhibitor suppresses the synergistic MMP-9 gene/protein expression, and attenuates the Smad2/3 phosphorylation. Further, as evident from Smad2/3 knockdown data, the Smad2/3 deficiency led to a marked decrease in synergistic induction of MMP-9 by TGF-β/TNF-α co-stimulation, indicating the essential role of Smad2/3 in this synergy. TGF-β-associated Smad2/3 activation played a central role in immune suppression by inducing Foxp3-positive regulatory T cells (49). We further demonstrate that TGF-β priming and Smad2/3 signaling drive synergy with TNF-α to promote MMP-9 expression in breast cancer cells. Most likely, TGF-β plays a dual role, depending on the milieu. Our findings are supported by a similar model showing that TGF-β, in presence of IL-6, promotes the pathogenic expansion of IL-17-producing Th17 cells, indirectly regulated by Smad2/3 signaling (50). This and the previous studies support that the role of TGF-β is very much milieu-dependent. The cellular effects of TGF-β are mediated via Smad and MAPK signaling pathways (51), and there is a complex interplay between these two types of signaling molecules. The MAPK pathways activated by TGF-β include the extracellular signal-regulated kinases, ERK1/2; the c-Jun N-terminal kinases, JNK1/2; and p38. Smad and MAPK pathways have functional interactions (52, 53) and our results show that the inhibition of p38 and ERK1/2 attenuates the synergy between TGF-β and TNF-α. Furthermore, Smad2/3 inhibition suppresses the p38 phosphorylation. The cells deficient in p38 exhibited a diminished synergy between TGF-β and TNF-α in promoting MMP-9 expression.

The interplay between states of chromatin and gene expression represents the key mechanisms that underly modulations in the expression of inflammatory mediators produced by various cells (54). TGF-β signals also via epigenetic mechanisms for the induction of complex gene expression programs (55). Since we identified a predominant involvement of TGF-β in cooperativity with TNF-α for amplifying MMP-9 gene expression, we speculated that TGF-β signaling implicated specific epigenetic alterations in this synergy. In this regard, inhibition of methyl transferases attenuated the synergy between TGF-β and TNF-α, resulting in suppression of MMP-9 expression. A comprehensive profiling of histone modifications identified H3K36me2 as the most significantly upregulated epigenetic chromatin mark in the breast cancer cells, in response to TGF-β/TNF-α co-stimulation. NSD2 is the primary histone methyltransferase that specifically dimethylates histone H3 at lysine 36 residue (H3K36me2) (56). siRNA-mediated knockdown of NSD2 in our breast cancer cell model significantly abolished the cooperativity between TGF-β and TNF-α for promoting MMP-9 expression, confirming the pivotal role of H3K36m2 chromatin active mark in this synergy. Our findings have some similarities to those reported by Sun et al., showing that TGF-β and high glucose induced significant promoter methylation changes at the H3K4 and H3K9 residues in rat mesangial cells that correlated with parallel increases in the expression of genes related to ECM accumulation and the pathogenesis of diabetic nephropathy (57). Since we found that the combined stimulation with TGF-β and TNF-α caused hyper-dimethylation at the histone H3K36 residue in breast cancer cells, we further addressed its mechanistic role in regulating the transcriptional activation of MMP-9, following cell stimulations with TGF-β, TNF-α, or TGF-β/TNF-α together. TGF-β and/or TNF-α stimulations of cells led to changes in the MMP-9 promoter histone methylation, as indicated by increased levels of transcriptionally permissive H3K36me2 chromatin mark, especially in cells co-stimulated with TGF-β and TNF-α. These data show that H3K36 dimethylation plays a critical role in regulating *MMP9* gene expression under the combined influence of TGF-β and TNF-α. These findings are in line with previous studies showing that TGF-β1 with high glucose leads to the enrichment of H3K4me1/2/3 marks at ECM-associated gene promoters in rat mesangial cells (57). Overall this synergistic effect mediated through the H3K36me2 epigenetic modification, underscores the importance of chromatin remodeling in the transcriptional regulation of genes critical to cancer metastasis. Notably, our study advances the understanding of MMP-9 regulation by highlighting the role of epigenetic modifications, a relatively unexplored frontier in MMP-9 research. The identification of H3K36me2 as a key epigenetic mark enriched in the presence of TGF-β and TNF-α provides a novel insight into the chromatin landscape alterations that facilitate MMP-9 expression. This discovery not only adds a new layer of complexity to our understanding of MMP-9 regulation but also opens new avenues for therapeutic interventions targeting epigenetic mechanisms. Notably, our study advances the understanding of MMP-9 regulation by highlighting the role of epigenetic modifications, a relatively unexplored frontier in MMP-9 research. The identification of H3K36me2 as a key epigenetic mark enriched in the presence of TGF-β and TNF-α provides a novel insight into the chromatin landscape alterations that facilitate MMP-9 expression. This discovery not only adds a new layer of complexity to our understanding of MMP-9 regulation but also opens new avenues for therapeutic interventions targeting epigenetic mechanisms. The clinical relevance of our study is underscored by the potential therapeutic implications of disrupting the TGF-β/TNF-α/MMP-9 axis. Given the pivotal role of MMP-9 in breast cancer metastasis, strategies aimed at inhibiting this pathway could offer a promising approach to limit cancer progression. The involvement of specific epigenetic modifications in this process further suggests the possibility of utilizing epigenetic modulators as part of a targeted therapy regimen, offering a novel strategy for the management of breast cancer. Our work also lays the groundwork for future research aimed at elucidating the complex interplay between cytokine signaling and epigenetic regulation in cancer. While we have demonstrated the synergistic effect of TGF-β and TNF-α on MMP-9 expression, the broader implications of this interaction within the intricate network of the TME warrant further investigation. Additionally, the potential for therapeutic targeting of this pathway highlights the need for the development of novel inhibitors that can selectively disrupt these signaling and epigenetic mechanisms. Our study not only sheds light on the regulatory mechanisms underlying MMP-9 expression in breast cancer but also opens new paths for the development of targeted therapeutic strategies. By unveiling the role of TGF-β and TNF-α in promoting MMP-9 expression through epigenetic modifications, we provide a foundation for future investigations into the complex molecular interactions that drive cancer metastasis. As we move forward, the insights gained from this study have the potential to significantly impact both our understanding and treatment of breast cancer.

In summary, our findings support that the synergistic interaction between TGF-β and TNF-α leads to MMP-9 overexpression in breast cancer cells via the mechanism hinged on Smad/p38/H3K36me2 mediated signaling, which may have significant pathophysiological implications for breast cancer invasion and metastasis.

### 4.1 Limitations of the study

In this study, we utilized cancer cell lines as our primary experimental models, which presents some notable limitations. One of the main constraints is the inherent difference between *in vitro* conditions and the complex *in vivo* environment of living organisms. Cell lines, while offering a controlled and reproducible platform, lack the intricate interactions found in whole organisms, including immune responses, blood flow, and the tumor microenvironment. This simplification may lead to an overestimation or underestimation of the therapeutic efficacy and biological behavior observed. Furthermore, cancer cell lines often originate from a single type of tumor, representing a narrow genetic and phenotypic spectrum of the disease, which may not accurately reflect the heterogeneity found in human cancers. This restricts the generalizability of our findings across different cancer types and patient populations. Another significant limitation is the difference between cancer cell lines and primary cancer cells derived directly from patients. Cancer cell lines, while providing a consistent and controllable model, often lack the heterogeneity and complex microenvironment found in primary tumors. This disparity can affect the relevance of our findings to real-world cancer biology. These limitations suggest that while our findings provide valuable insights, they should be interpreted with caution, and future research should aim to include primary cancer cells to better mimic the tumor microenvironment and provide more clinically relevant insights. Further validation in animal models and clinical settings is essential to confirm the therapeutic potential and safety of the investigated compounds.

## Data Availability

The original contributions presented in the study are included in the article/**Supplementary Material**. Further inquiries can be directed to the corresponding authors.

## References

[1] Lei S Zheng R Zhang S Wang S Chen R Sun K. Global patterns of breast cancer incidence and mortality: A population-based cancer registry data analysis from 2000 to 2020. Cancer Commun (Lond). (2021) 41:1183–94. doi: 10.1002/cac2.12207 PMC8626596 34399040

[2] Arnold M Morgan E Rumgay H Mafra A Singh D Laversanne M. Current and future burden of breast cancer: Global statistics for 2020 and 2040. Breast (Edinburgh Scotland). (2022) 66:15–23. doi: 10.1016/j.breast.2022.08.010 36084384 PMC9465273

[3] Azadnajafabad S Saeedi Moghaddam S Keykhaei M Shobeiri P Rezaei N Ghasemi E. Expansion of the quality of care index on breast cancer and its risk factors using the global burden of disease study 2019. Cancer Med. (2023) 12:1729–43. doi: 10.1002/cam4.4951 PMC9883412 35770711

[4] Khaled N Bidet Y. New insights into the implication of epigenetic alterations in the EMT of triple negative breast cancer. Cancers. (2019) 11(4):1–21. doi: 10.3390/cancers11040559 PMC6521131 31003528

[5] Cruceriu D Baldasici O Balacescu O Berindan-Neagoe I. The dual role of tumor necrosis factor-alpha (TNF-α) in breast cancer: molecular insights and therapeutic approaches. Cell Oncol (Dordrecht). (2020) 43:1–18. doi: 10.1007/s13402-019-00489-1 PMC12990688 31900901

[6] Segovia-Mendoza M Morales-Montor J. Immune tumor microenvironment in breast cancer and the participation of estrogen and its receptors in cancer physiopathology. Front Immunol. (2019) 10:348. doi: 10.3389/fimmu.2019.00348 30881360 PMC6407672

[7] Hassan G Seno M. Blood and cancer: cancer stem cells as origin of hematopoietic cells in solid tumor microenvironments. Cells. (2020) 9(5):1–15. doi: 10.3390/cells9051293 PMC7290570 32455995

[8] King J Mir H Singh S. Association of cytokines and chemokines in pathogenesis of breast cancer. Prog Mol Biol Trans Sci. (2017) 151:113–36. doi: 10.1016/bs.pmbts.2017.07.003 29096891

[9] Nascimento C Ferreira F. Tumor microenvironment of human breast cancer, and feline mammary carcinoma as a potential study model. Biochim Biophys Acta Rev Cancer. (2021) 1876:188587. doi: 10.1016/j.bbcan.2021.188587 34237352

[10] Radisky ES Raeeszadeh-Sarmazdeh M Radisky DC. Therapeutic potential of matrix metalloproteinase inhibition in breast cancer. J Cell Biochem. (2017) 118:3531–48. doi: 10.1002/jcb.26185 PMC5621753 28585723

[11] Wu ZS Wu Q Yang JH Wang HQ Ding XD Yang F. Prognostic significance of MMP-9 and TIMP-1 serum and tissue expression in breast cancer. Int J Cancer. (2008) 122:2050–6. doi: 10.1002/ijc.23337 18172859

[12] Joseph C Alsaleem M Orah N Narasimha PL Miligy IM Kurozumi S. Elevated MMP9 expression in breast cancer is a predictor of shorter patient survival. Breast Cancer Res Treat. (2020) 182:267–82. doi: 10.1007/s10549-020-05670-x PMC7297818 32445177

[13] Zhou XL Fan W Yang G Yu MX. The clinical significance of PR, ER, NF- κ B, and TNF- α in breast cancer. Dis Markers. (2014) 2014:494581. doi: 10.1155/2014/494581 24864130 PMC4017837

[14] Park KS Mok JW Ko HE Tokunaga K Lee MH. Polymorphisms of tumour necrosis factors A and B in breast cancer. Eur J immunogenetics: Off J Br Soc Histocompatibility Immunogenetics. (2002) 29:7–10. doi: 10.1046/j.0960-7420.2001.00260.x 11841482

[15] Liu W Lu X Shi P Yang G Zhou Z Li W. TNF-α increases breast cancer stem-like cells through up-regulating TAZ expression via the non-canonical NF-κB pathway. Sci Rep. (2020) 10:1804. doi: 10.1038/s41598-020-58642-y 32019974 PMC7000832

[16] Eftekhari R Esmaeili R Mirzaei R Bidad K de Lima S Ajami M. Study of the tumor microenvironment during breast cancer progression. Cancer Cell Int. (2017) 17:123. doi: 10.1186/s12935-017-0492-9 29299026 PMC5741925

[17] Wolczyk D Zaremba-Czogalla M Hryniewicz-Jankowska A Tabola R Grabowski K Sikorski AF. TNF-α promotes breast cancer cell migration and enhances the concentration of membrane-associated proteases in lipid rafts. Cell Oncol (Dordrecht). (2016) 39:353–63. doi: 10.1007/s13402-016-0280-x PMC4972855 27042827

[18] Hong OY Jang HY Lee YR Jung SH Youn HJ Kim JS. Inhibition of cell invasion and migration by targeting matrix metalloproteinase-9 expression via sirtuin 6 silencing in human breast cancer cells. Sci Rep. (2022) 12:12125. doi: 10.1038/s41598-022-16405-x 35840633 PMC9287314

[19] Barnard JA Lyons RM Moses HL. The cell biology of transforming growth factor beta. Biochim Biophys Acta. (1990) 1032(1):79–87. doi: 10.1016/0304-419x(90)90013-q 2194569

[20] Roberts AB Wakefield LM. The two faces of transforming growth factor beta in carcinogenesis. Proc Natl Acad Sci United States America. (2003) 100:8621–3. doi: 10.1073/pnas.1633291100 PMC166359 12861075

[21] Liu ZW Zhang YM Zhang LY Zhou T Li YY Zhou GC. Duality of interactions between TGF-β and TNF-α During tumor formation. Front Immunol. (2021) 12:810286. doi: 10.3389/fimmu.2021.810286 35069596 PMC8766837

[22] Kong FM Anscher MS Murase T Abbott BD Iglehart JD Jirtle RL. Elevated plasma transforming growth factor-beta 1 levels in breast cancer patients decrease after surgical removal of the tumor. Ann Surg. (1995) 222:155–62. doi: 10.1097/00000658-199508000-00007 PMC1234773 7543740

[23] Dalal BI Keown PA Greenberg AH. Immunocytochemical localization of secreted transforming growth factor-beta 1 to the advancing edges of primary tumors and to lymph node metastases of human mammary carcinoma. Am J Pathol. (1993) 143(2):381–9. PMC1887030 8393616

[24] Dong H Diao H Zhao Y Xu H Pei S Gao J. Overexpression of matrix metalloproteinase-9 in breast cancer cell lines remarkably increases the cell Malignancy largely via activation of transforming growth factor beta/SMAD signalling. Cell proliferation. (2019) 52:e12633. doi: 10.1111/cpr.12633 31264317 PMC6797518

[25] Sun Y Zhou QM Lu YY Zhang H Chen QL Zhao M. Resveratrol inhibits the migration and metastasis of MDA-MB-231 human breast cancer by reversing TGF-β1-induced epithelial-mesenchymal transition. Molecules. (2019) 24(6):1–16. doi: 10.3390/molecules24061131 PMC6471699 30901941

[26] Mo N Li ZQ Li J Cao YD. Curcumin inhibits TGF-β1-induced MMP-9 and invasion through ERK and Smad signaling in breast cancer MDA- MB-231 cells. Asian Pacific J Cancer prevention: APJCP. (2012) 13:5709–14. doi: 10.7314/APJCP.2012.13.11.5709 23317243

[27] Ridner SH Dietrich MS Sonis ST Murphy B. Biomarkers associated with lymphedema and fibrosis in patients with cancer of the head and neck. Lymphatic Res Biol. (2018) 16:516–24. doi: 10.1089/lrb.2017.0074 PMC6306661 30484735

[28] Kamitani S Yamauchi Y Kawasaki S Takami K Takizawa H Nagase T. Simultaneous stimulation with TGF-β1 and TNF-α induces epithelial mesenchymal transition in bronchial epithelial cells. Int Arch Allergy Immunol. (2011) 155:119–28. doi: 10.1159/000318854 21196756

[29] Han Y-P Tuan T-L Hughes M Wu H Garner WL. Transforming growth factor-β- and tumor necrosis factor-α-mediated induction and proteolytic activation of MMP-9 in human skin*. J Biol Chem. (2001) 276:22341–50. doi: 10.1074/jbc.M010839200 PMC2651823 11297541

[30] Stuelten CH DaCosta Byfield S Arany PR Karpova TS Stetler-Stevenson WG Roberts AB. Breast cancer cells induce stromal fibroblasts to express MMP-9 via secretion of TNF-alpha and TGF-beta. J Cell Sci. (2005) 118:2143–53. doi: 10.1242/jcs.02334 15855236

[31] Liao SJ Luo J Li D Zhou YH Yan B Wei JJ. TGF-β1 and TNF-α synergistically induce epithelial to mesenchymal transition of breast cancer cells by enhancing TAK1 activation. J Cell communication Signaling. (2019) 13:369–80. doi: 10.1007/s12079-019-00508-8 PMC6732143 30739244

[32] Kochumon S Arefanian H Sindhu S Thomas R Jacob T Al-Sayyar A. Expression of steroid receptor RNA activator 1 (SRA1) in the adipose tissue is associated with TLRs and IRFs in diabesity. Cells. (2022) 11(24):1–18. doi: 10.3390/cells11244007 PMC9776802 36552771

[33] Al-Mulla F Bitar MS Thiery JP Zea TT Chatterjee D Bennett L. Clinical implications for loss or diminution of expression of Raf-1 kinase inhibitory protein and its phosphorylated form in ductal breast cancer. Am J Cancer Res. (2013) 3(5):446–64. PMC3816965 24224123

[34] Al-Roub A Akhter N Al-Rashed F Wilson A Alzaid F Al-Mulla F. TNFα induces matrix metalloproteinase-9 expression in monocytic cells through ACSL1/JNK/ERK/NF-kB signaling pathways. Sci Rep. (2023) 13:14351. doi: 10.1038/s41598-023-41514-6 37658104 PMC10474281

[35] Al-Rashed F Haddad D Al Madhoun A Sindhu S Jacob T Kochumon S. ACSL1 is a key regulator of inflammatory and macrophage foaming induced by short-term palmitate exposure or acute high-fat feeding. iScience. (2023) 26:107145. doi: 10.1016/j.isci.2023.107145 37416456 PMC10320618

[36] Liu K Newbury PA Glicksberg BS Zeng WZD Paithankar S Andrechek ER. Evaluating cell lines as models for metastatic breast cancer through integrative analysis of genomic data. Nat Commun. (2019) 10:2138. doi: 10.1038/s41467-019-10148-6 31092827 PMC6520398

[37] Cai S Zheng J Song H Wu H Cai W. Relationship between serum TGF- β 1, MMP-9 and IL-1β and pathological features and prognosis in breast cancer. Front Genet. (2022) 13:1095338. doi: 10.3389/fgene.2022.1095338 36712861 PMC9877302

[38] Kim H-S Luo L Pflugfelder SC Li D-Q. Doxycycline inhibits TGF-β1–induced MMP-9 via smad and MAPK pathways in human corneal epithelial cells. Invest Ophthalmol Visual Sci. (2005) 46:840–8. doi: 10.1167/iovs.04-0929 15728539

[39] Massagué J. TGFβ signalling in context. Nat Rev Mol Cell Biol. (2012) 13(10):616–30. doi: 10.1038/nrm3434 PMC4027049 22992590

[40] Li Z Zhang X Xie S Liu X Fei C Huang X. H3K36me2 methyltransferase NSD2 orchestrates epigenetic reprogramming during spermatogenesis. Nucleic Acids Res. (2022) 50:6786–800. doi: 10.1093/nar/gkac533 PMC9262605 35736136

[41] Zhao LH Li Q Huang ZJ Sun MX Lu JJ Zhang XH. Identification of histone methyltransferase NSD2 as an important oncogenic gene in colorectal cancer. Cell Death Dis. (2021) 12:974. doi: 10.1038/s41419-021-04267-6 34671018 PMC8528846

[42] Chen R Chen Y Zhao W Fang C Zhou W Yang X. The role of methyltransferase NSD2 as a potential oncogene in human solid tumors. OncoTargets Ther. (2020) 13:6837–46. doi: 10.2147/OTT.S259873 PMC7367929 32764971

[43] McGowan PM Duffy MJ. Matrix metalloproteinase expression and outcome in patients with breast cancer: analysis of a published database. Ann oncology: Off J Eur Soc Med Oncol. (2008) 19:1566–72. doi: 10.1093/annonc/mdn180 18503039

[44] Merdad A Karim S Schulten HJ Dallol A Buhmeida A Al-Thubaity F. Expression of matrix metalloproteinases (MMPs) in primary human breast cancer: MMP-9 as a potential biomarker for cancer invasion and metastasis. Anticancer Res. (2014) 34(3):1355–66. 24596383

[45] Drabsch Y ten Dijke P. TGF-β Signaling in breast cancer cell invasion and bone metastasis. J Mammary Gland Biol Neoplasia. (2011) 16:97–108. doi: 10.1007/s10911-011-9217-1 21494783 PMC3095797

[46] Augoff K Hryniewicz-Jankowska A Tabola R Stach K. MMP9: A tough target for targeted therapy for cancer. Cancers. (2022) 14(7):1–28. doi: 10.3390/cancers14071847 PMC8998077 35406619

[47] Han YP Tuan TL Hughes M Wu H Garner WL. Transforming growth factor-beta - and tumor necrosis factor-alpha -mediated induction and proteolytic activation of MMP-9 in human skin. J Biol Chem. (2001) 276:22341–50. doi: 10.1074/jbc.M010839200 PMC2651823 11297541

[48] Wrighton KH Lin X Feng XH. Phospho-control of TGF-beta superfamily signaling. Cell Res. (2009) 19:8–20. doi: 10.1038/cr.2008.327 19114991 PMC2929013

[49] Yoshimura A Muto G. TGF-β function in immune suppression. Curr topics Microbiol Immunol. (2011) 350:127–47. doi: 10.1007/82_2010_87 20680806

[50] Qin H Wang L Feng T Elson CO Niyongere SA Lee SJ. TGF-beta promotes Th17 cell development through inhibition of SOCS3. J Immunol. (2009) 183:97–105. doi: 10.4049/jimmunol.0801986 19535626 PMC2851540

[51] Leivonen SK Kähäri VM. Transforming growth factor-beta signaling in cancer invasion and metastasis. Int J Cancer. (2007) 121:2119–24. doi: 10.1002/ijc.23113 17849476

[52] Javelaud D Mauviel A. Crosstalk mechanisms between the mitogen-activated protein kinase pathways and Smad signaling downstream of TGF-beta: implications for carcinogenesis. Oncogene. (2005) 24:5742–50. doi: 10.1038/sj.onc.1208928 16123807

[53] Hanafusa H Ninomiya-Tsuji J Masuyama N Nishita M Fujisawa J Shibuya H. Involvement of the p38 mitogen-activated protein kinase pathway in transforming growth factor-beta-induced gene expression. J Biol Chem. (1999) 274:27161–7. doi: 10.1074/jbc.274.38.27161 10480932

[54] Shanmugam MK Sethi G. Role of epigenetics in inflammation-associated diseases. Subcell Biochem. (2013) 61:627–57. doi: 10.1007/978-94-007-4525-4_27 23150270

[55] Massagué J. TGFβ signalling in context. Nature reviews. Mol Cell Biol. (2012) 13(10):616–30. doi: 10.1038/nrm3434"10.1038/nrm3434 PMC4027049 22992590

[56] Hanley RP Nie DY Tabor JR Li F Sobh A Xu C. Discovery of a potent and selective targeted NSD2 degrader for the reduction of H3K36me2. J Am Chem Soc. (2023) 145:8176–88. doi: 10.1021/jacs.3c01421 PMC10116495 36976643

[57] Sun G Reddy MA Yuan H Lanting L Kato M Natarajan R. Epigenetic histone methylation modulates fibrotic gene expression. J Am Soc Nephrology: JASN. (2010) 21:2069–80. doi: 10.1681/ASN.2010060633 PMC3014020 20930066

