# Supplementary material for: TGF-β and TNF-α interaction promotes the expression of MMP-9 through H3K36 dimethylation: implications in breast cancer metastasis
Source: Front Immunol. 2024 Sep 16;15:1430187. doi: 10.3389/fimmu.2024.1430187 (PMC11439675; doi:10.3389/fimmu.2024.1430187)
Supplement: Supplementary file 1 [file DataSheet1.pdf]

Supplementary Material

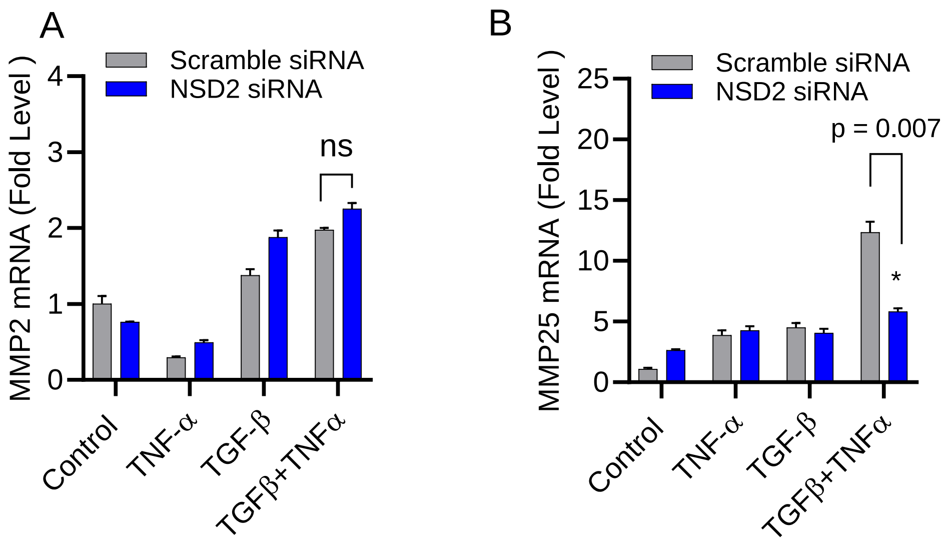

Figure S1A-B. NSD2 histone methyltransferase knockdown effectively suppresses MMP-25 expression co-induced by TGF-β /TNF-α. MDA-MB-231 cells were transfected with either NSD2 siRNA (20 nM) or scrambled siRNA (20 nM) as described in the Materials and Methods section. (**A-B**) MDA-MB-231 cells deficient with NSD2 were incubated with vehicle, TGF-β, TNF-α or TGF-β/TNF-α. Cells and supernatants were collected and MMP-2 and MMP-25 mRNA were measured by real-time RT-PCR. All data are presented as mean ±SEM (n=3) and compared between groups using unpaired t-test. *$p < 0.05$.

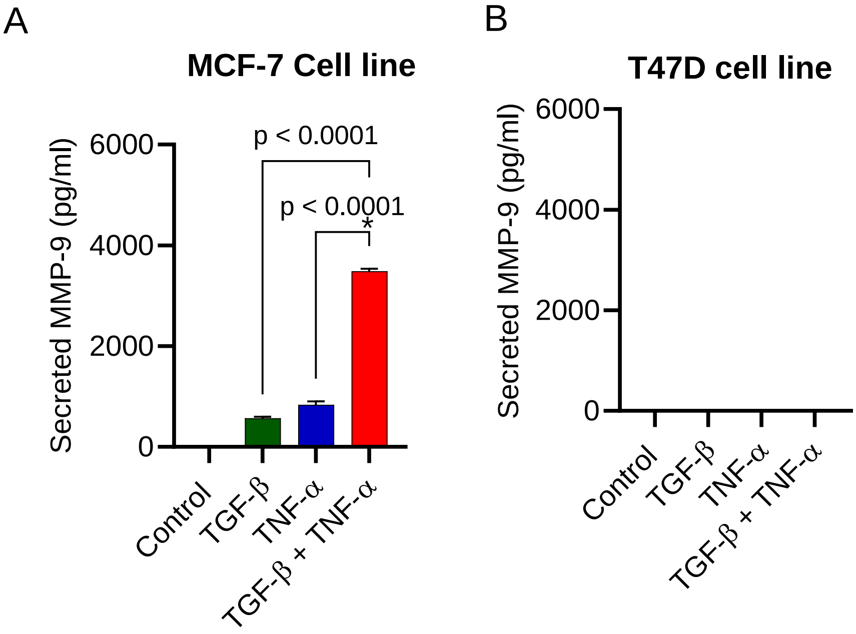

Figure S2 A & B. TGF-β/TNF-α cooperatively increased MMP-9 expression in human MCF-7 breast cancer cells. MCF-7 or T47D cells were cultured in 12-well plates with a cell concentration set at 0.3×10^6 cells per well. Cells were subjected to a 24 h treatment with vehicle, TGF-β (20 ng/mL), TNF-α (10 ng/mL), or TGF-β/TNF-α. Supernatants were collected following incubation period. (**A-B**) The concentration of secreted MMP-9 protein in the culture media was assessed by ELISA. Data were presented as mean ± SEM (n = 3) and compared using one-way ANOVA with Tukey's test. ns: non-significant, $*p \leq 0.05$.

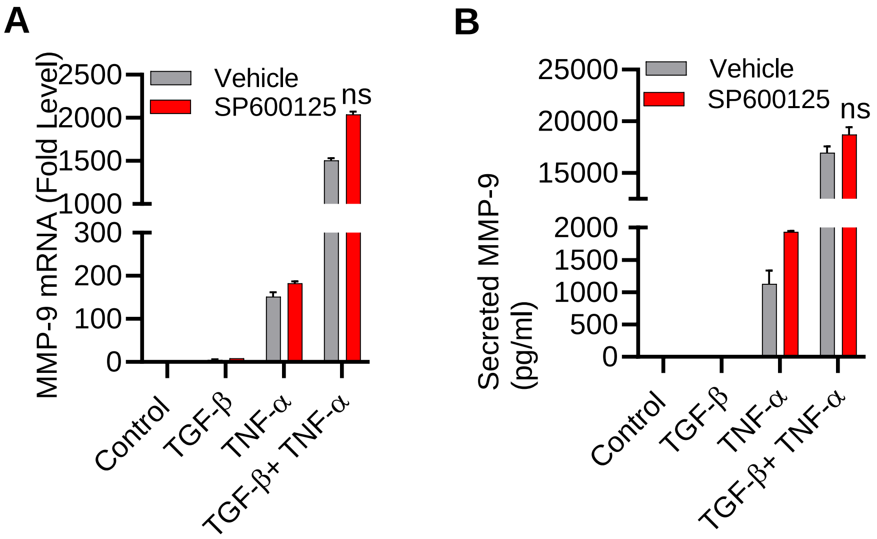

Figure S3A-B. MDA-MB-231 breast cancer cells were pretreated for 1 h with JNK (SP600125; 10 μM) inhibitor and then incubated with TGF-β, TNFα or TGF-β/TNF-α for 24 h. Both cells and their culture media were collected. MMP9 mRNA and protein expression were determined. All data is presented as the mean ± SEM (n= 3). $*p < 0.05$

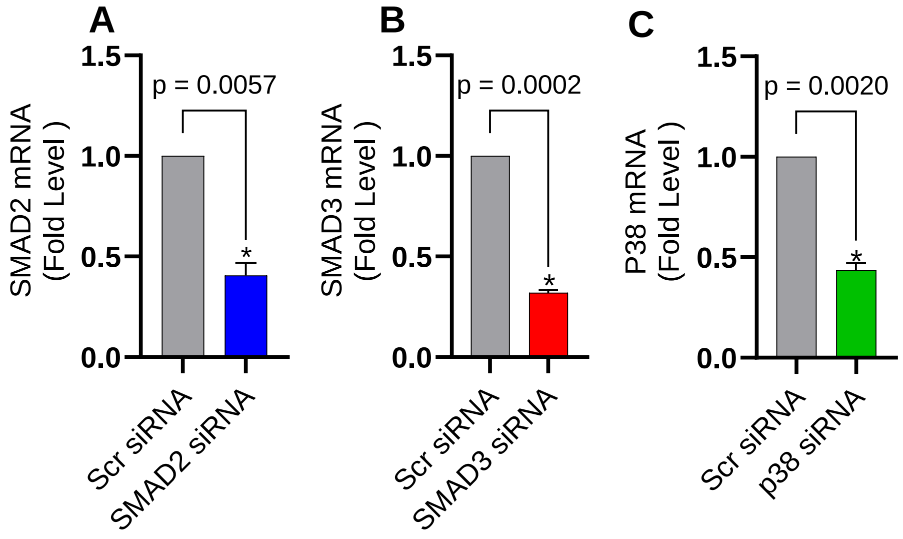

Figure S4. (**A-C**) Transfection of MDA-MB-231 cells was carried out using SMAD2 siRNA (20 nM), SMAD3 siRNA (20 nM), p38 siRNA (20 nM), or scrambled siRNA (20 nM) following the procedures detailed in the Materials and Methods section. Measurement of SMAD 2, SMAD 3 or p38 mRNA was carried out through real-time RT-PCR after a 36-hour interval. All data is presented as the mean ± SEM (n= 3). *$p < 0.05$.

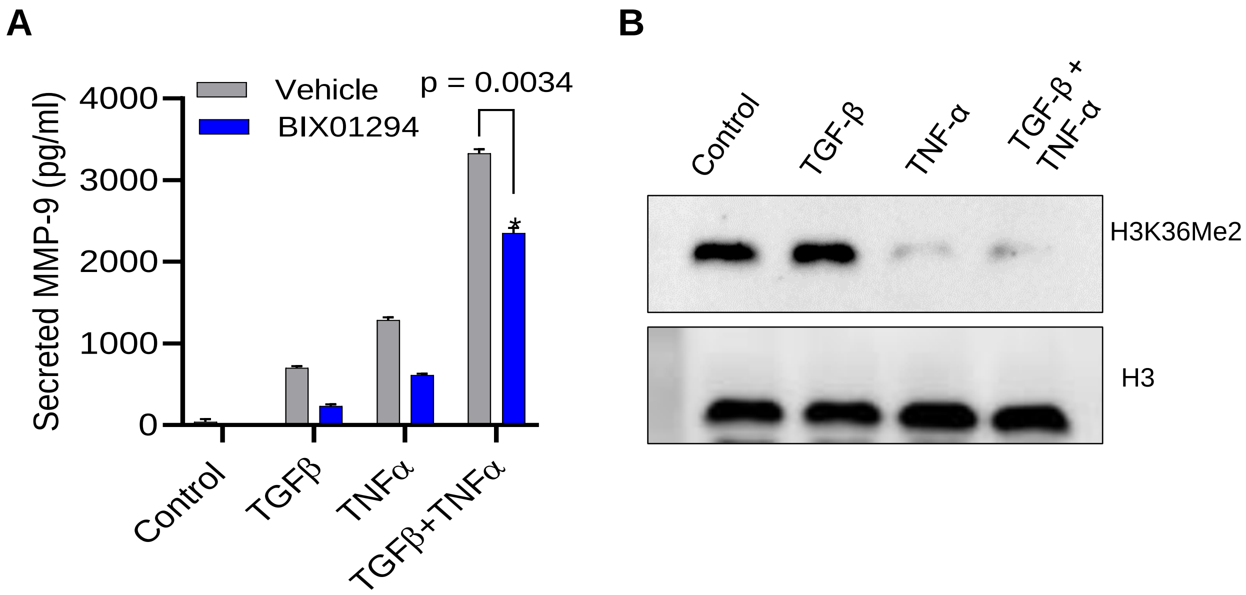

Figure S5 A-B. TGF-β/TNF-α synergistically increased MMP-9 production in MCF-7 cells through histone methylation, but not specifically via H3K36 Dimethylation. MCF-7 breast cancer cells were treated with BIX 01294 (10µM) for 1 h and then treated with TGF-β/ TNF-α for 24 h. Culture supernatant were collected. (**A**) Secreted MMP9 protein in culture media was determined by ELISA. (**B**) After a 3-hour exposure to the vehicle, TGF-β, TNF-α, or TGF-β/ TNF-α, MCF-7 cells were analyzed for histone methylation levels using Western blot. Data are presented as mean ± SEM values (n=3) and compared between groups using unpaired t-test. *p < 0.05.

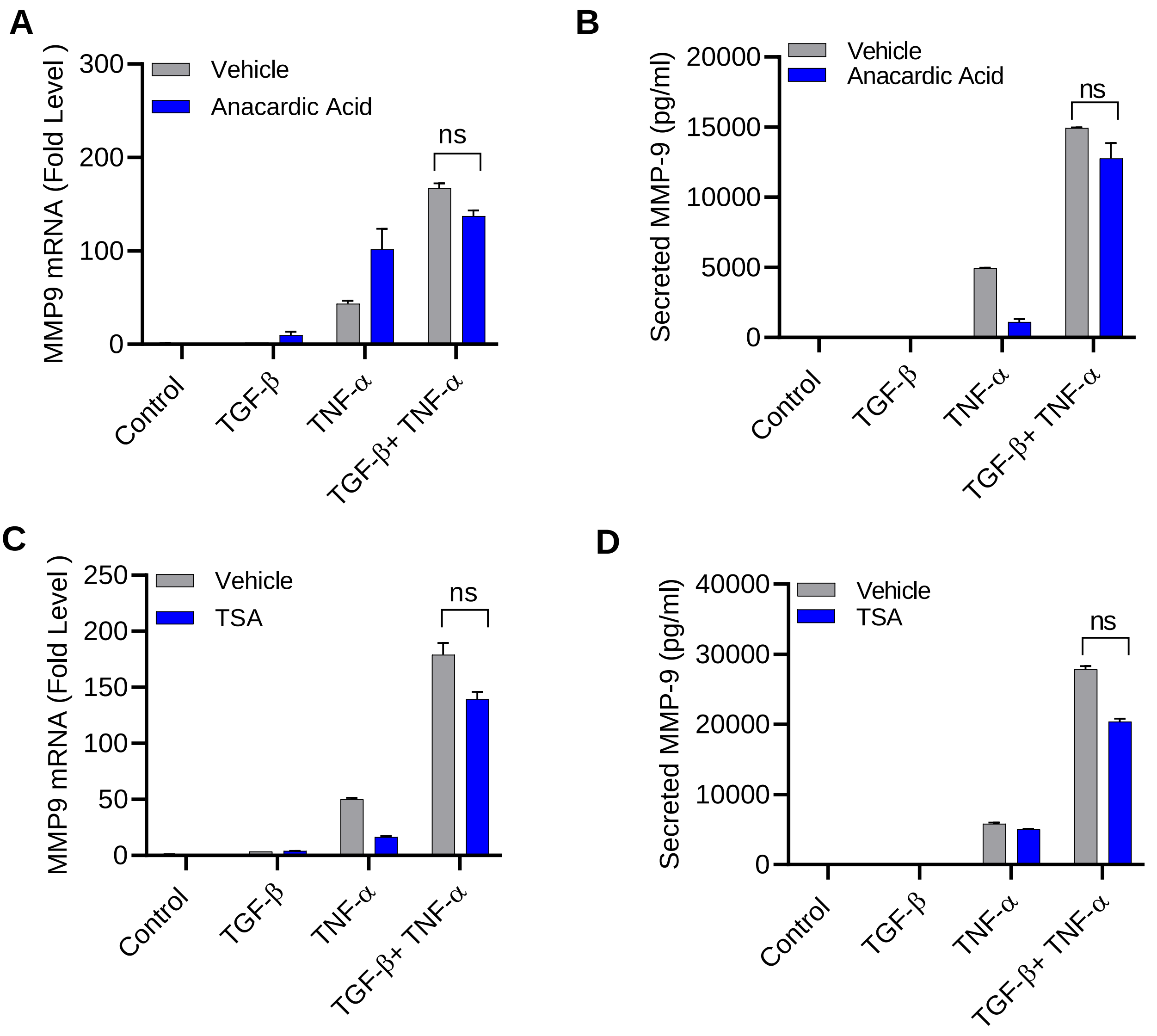

Figure S6. The synergistic expression of MMP-9 is independent of histone acetylation. Anacardic acid or TSA did not affect the synergistic expression of MMP-9 induced by TGF-β/TNF-α. Prior to exposure to vehicle, TGF-β, TNF-α, or TGF-β/TNF-α, cells were subjected to anacardic acid treatment at a concentration of 4 μM for 1 h. Both cells and the culture supernatant were harvested. (**A**) Total RNA was extracted, and MMP-9 mRNA was quantified by RT-PCR. (**B**) Secreted MMP-9 protein in culture media was determined by ELISA. (**C** and **D**) Cells were treated with TSA (25 nM) for 1 h, followed by a 24-hour stimulation with vehicle, TGF-β, TNF-α, or TGF-β/TNF-α.MMP-9 mRNA and protein were determined. All data are presented as the mean ± SEM (n= 3). *$p < 0.05$.

Supplementary Methodology File

Materials and Methods

Human breast cancer tumor samples and immunohistochemistry
Tissue microarray (TMA) slides (Breast carcinoma with matched metastatic carcinoma or breast tissue microarray, containing 48 cases of breast carcinoma, 36 metastatic carcinoma (35 matched with their primary breast carcinoma), 12 adjacent or adjacent normal breast tissue; BRM961a) were obtained from TissueArray.Com LLC 1(5885 Crabbs Branch Way, Derwood, MD 20855, USA). Characteristics of the breast cancer patients and their tumor clinicopathology were described in Table S1. Immunohistochemical staining was performed on 96 breast cancer tissues of 48 patients with metastatic breast cancer, arranged in tissue microarrays as described earlier [1, 2]. Briefly, TMA slides were deparaffinized in xylene and rehydrated through descending grades of ethanol (100%, 95%, and 75%) to water. Antigen retrieval was performed by placing slides in target retrieval solution (pH6.0; Dako, Glostrup, Denmark) under a pressure cooker boiling for 8 min and cooling for 15 min. After washing in PBS, endogenous peroxidase activity was blocked with 3% H2O2 for 30 min and non-specific antibody binding was blocked with 5% nonfat milk for 1hr and 1% bovine serum albumin (BSA) solution for 1hr. Slides were treated overnight with primary antibodies (anti-TNF-α, 1:200, Cat# ab1793, Abcam, Cambridge, UK; anti-MMP-9, 1:200, Cat# ab38898, Abcam, Cambridge, UK; mouse anti-human TGF-β, 1:500, Cat# MCA 797, Bio-Rad, Hercules, California, USA) at room temperature using dilutions as recommended by manufacturers. After washing with PBS (were% Tween), slides were incubated for 1hr with a secondary antibody conjugated with HRP polymer chain (EnVision Kit, Dako, Glostrup, Denmark), and color was developed using 3,3'-diaminobenzidine chromogen substrate. Specimens were washed in running tap water, lightly counterstained with Harris hematoxylin, dehydrated through ascending grades of ethanol (75%, 95%, and 100%), cleared in xylene, and finally mounted in dibutyl phthalate xylene (DPX).
Slide scanning and scoring
The stained sample slides were evaluated under the light microscope by trained pathologist or researcher as described earlier[2]. Semi-quantitative scoring systems are widely used to convert subject perception of IHC-marker expression into (semi)quantitative data, which is then used for statistical analyses and establishing of the conclusions. The existing clinical scoring process is based on two characteristics: overall staining intensity and the proportion of tissue or cells stained. The overall score of the staining intensity typically has four categories: negative (0), weak (1), moderate (2), and strong (3). H-score, Allred-score, and Immunoreactive score are considered as a “gold standard” of combined scoring system in IHC data evaluation and presentation. All these scoring systems use different categories for the proportion of tissues or cells stained[2]. The H-score method takes into account both the area and intensity of staining to generate values between 0-300 using the following formula: Σ (1 × % cells staining weakly positive) + (2 × % cells staining moderately positive) + (3 × % cells staining strongly positive).

Quartile assignment based on TGF-β and TNF-α H-scores
For statistical comparisons, the human breast cancer tissue samples were categorized into 4 quartiles depending on TGF-β and TNF-α H-score values: first quartile (Low TNF-α- Low TGF-β), contained breast cancer tissues, which have H-score values: TNF-α <169.7- and TGF-β <223.2 ; second quartile (Low TNF-α- High TGF-β), contained breast cancer tissues, which have H-score values: TNF-α: <169.7 and TGF-β: ≥223.2; third quartile (High TNF-α- Low TGF-β), contained breast cancer tissues, which have H-score values: TNF-α: ≥169.7 and TGF-β: <223.2; fourth quartile (High TNF-α- High TGF-β), contained breast cancer tissues, which have H-score values: TNF-α: ≥169.7 and TGF-β: ≥223.2. All quartiles are shown in the form of scatter plot graphs for TGF- β and TNF- α (Fig. 2A -B).

Cell Culture
The MDA-MB-231 is a well-established cell line model for triple-negative breast cancer. It is commonly used to identify genes and pathways that are linked to specific metastatic sites. Human MDA-MB-231 cells were purchased from American Type Culture Collection (ATCC. Manassas, VA, USA), and then grown in

DMEM culture medium (Gibco, Thermo Fisher Scientific, Waltham, MA USA), supplemented with 10% of fetal bovine serum (Gibco, Thermo Fisher Scientific, Waltham, MA, USA), 2mM glutamine (Gibco, Thermo Fisher Scientific, Waltham, MA, USA), 50 U/mL penicillin and 50 μg/mL streptomycin (P/S; Gibco, Thermo Fisher Scientific, Waltham, MA, USA) and incubated at 37 °C (with humidity) in 5% CO2.

Cell Stimulation

MDA-MB-231 cells were plated in a 12-well plate (Costar, Corning Incorporated, Corning, NY, USA) at 0.3×106 cells/well concentration unless otherwise indicated. After 48-hour incubation, cells were treated with vehicle (0.1%BSA) , TGF-β (20 ng/mL; 240-B, R&D Systems, Minneapolis, MN, USA), TNF-α (10 ng/mL; 210-TA, R&D Systems, Minneapolis, MN, USA) or TGF-β/TNF-α for 24 h at 37 °C. Cells were harvested for RNA isolation and conditioned media was collected for measuring secreted MMP-9. For Smad 2/3 and P38 signaling pathways, cells were preincubated with different cell signaling pathway inhibitors for 1 h under cell culture conditions (SB203580 (10 μM; 559389, Sigma-Aldrich, Merck KGaA, Darmstadt, Germany), a p38 inhibitor; U0126 (10 μM; tlrl-u0126, InvivoGen, San Diego, CA, USA), a MEK1/2 inhibitor; SP600125 (10 μM; 420119, Sigma-Aldrich, Merck KGaA, Darmstadt, Germany), a JNK inhibitor; IN-1130 (10 μM, SML1403-25MG, Sigma) Smad inhibitor and LY364947 (10 μM, L6293-25MG, Sigma) a TGFβ-R signaling pathway inhibitor. Cells were then treated with vehicle, TGFβ , TNF-α or TGF-β/TNF-α for 24 hours and harvested for RNA isolation and conditioned media were collected for measuring secreted MMP-9.

Real-Time Quantitative PCR

Total RNA was extracted from MDA-MB-231 cells using RNeasy Mini Kit (Qiagen, Valencia, CA, USA) as per the manufacturer's instructions. The cDNA was synthesized using 1 μg of total RNA using a high-capacity cDNA reverse transcription kit (Applied Biosystems, Foster City, CA, USA)[3]. Real-time PCR was performed on a QuantStudio™ 5 Real-Time PCR System (Applied Biosystems, Foster City, CA, USA) using a TaqManTM Gene Expression Master Mix (Applied Biosystems, Foster City, CA, USA). Each reaction contained 50ng cDNA that was amplified with Inventoried TaqMan Gene Expression Assay products (MMP9: Hs00957562_m1, SMAD2: Hs00998187_m1, SMAD3: Hs00969210_m1, P38: Hs01051152_m1, NSD2: Hs00983720_m1, MMP-2, Assay ID: Hs01548727_m1, MMP25, Assay ID: Hs01554789_m1, GAPDH: Hs03929097_g1). The threshold cycle (Ct) values were normalized to the housekeeping gene GAPDH and the amounts of target mRNA relative to the control were calculated using the 2−ΔΔCt method [4, 5]. Relative mRNA expression was expressed as a fold expression over an average of control gene expression. The expression level in the control treatment was assumed to be 1. Values are presented as mean ± SEM. Results were analyzed statistically; $p < 0.05$ was considered significant.

MMP-9 Determination

Secreted MMP-9 protein in supernatants of MDA-MB-231 cells stimulated with vehicle, TGF-β, TNFα or TGF-β/TNF-α was quantified using Human MMP-9 Quantikine ELISA Kit (DMP900, R&D Systems, Minneapolis, MN, USA) following the manufacturer's instructions[6].

Western Blotting

MDA-MB-231 cells were treated with TGFβ/ TNFα and were harvested after incubation. The cells were then incubated for 30 minutes with lysis buffer (10X Lysis Buffer, Cell Signaling, Danvers, MA, USA). The lysates were centrifuged at 14,000 g for 10 minutes and the protein supernatants were collected. Protein concentrations were measured by Quickstart Bradford Dye Reagent, 1x Protein Assay Kit (Bio-Rad Laboratories, Inc, CA). Protein (20 μg) samples were mixed with loading buffer, heated for 5 min at 95°C, and resolved by 12% SDS-PAGE. Cellular proteins were transferred to an Immuno-Blot Polyvinylidene difluoride (PVDF) membrane (Bio-Rad Laboratories, Hercules, CA, USA) by electroblotting[7]. The membranes were then blocked with 5% non-fat milk in PBS for 1 hour, followed by incubation with primary antibodies against H3K36Me2 (catalog no. 2901), Histone H3 (catalog no. 4499), p-p38 (catalog no. 9216), p38 (catalog no. 9212, p-SMAD2 (catalog no. 3108), SMAD2 (catalog no. 5339), p-SMAD3 (catalog no.9520) and SMAD3 (catalog no. 9523) in 1:1000 dilution at 4 °C overnight. All primary antibodies were purchased from Cell Signaling (Cell Signaling Technology Inc., Danvers, MA, USA). The blots were then washed three times with TBS-T and incubated for 2 h with HRP-conjugated secondary antibody (Promega, Madison, WI, USA). Immunoreactive bands were developed using Amersham ECL Plus Western Blotting

Detection System (GE Healthcare, Chicago, IL, USA) and visualized by Molecular Imager® VersaDocTM MP Imaging Systems (Bio-Rad Laboratories, Hercules, CA, USA)[8].

Small Interfering RNA (siRNA) Transfection

Transient transfection of MDA-MB-231 cells was done using Lipofectamine RNAiMAX reagent (Themo Fischer, USA) following the manufacturer's instructions and was transfected separately with Smad2-siRNA (20 nM; s8397, Thermo Fischer, USA), Smad3-siRNA (20 nM; s8401, Thermo Fischer, USA), p38-siRNA (20 nM; s3538, Thermo Fischer, USA), NSD2 (20 nM; s526860, Thermo Fischer, USA) and scramble (negative control) siRNA (20 nM; 4390843, Thermo Fischer, USA). After 48 hours of transfection, cells were treated with vehicle, TGF-β, TNF-α or TGFβ /TNFα and incubated for 24 hours. Cells and conditioned medium were harvested for RNA isolation and ELISA. The knockdown of Smad 2/3, NSD2, and P38 pathways was assessed using Real-Time PCR gene-specific primer probes.

Zymography

MDA-MB-231 cells were incubated with TGFβ /TNFα. After incubation for 24 hours, conditioned media were collected and mixed with Zymogram sample buffer, BioRad (62.5mM Tris-HCl, pH.6.8, 25% glycerol, 4%SDS and 0.01% bromophenol blue) and loaded on to a 10% polyacrylamide gel with gelatin (10% Ready Gel® Zymogram Gel , Biorad) for electrophoresis. The gel was incubated with renaturing buffer, BioRad (2.5% Triton X-100) for 1 hour at room temperature and incubated with zymogram developing buffer, BioRad (50mM Tris-HCl, pH 7.5, 200mM NaCl and 5mM $CaCl_2$) for 24 hours at 37°C. Gels were stained with staining solution (0.5% Coomassie Brilliant Blue R-250, 40% Methanol, 10% Acetic Acid) for 2 hours and then de-stained with de-staining solution (40% Methanol, 10% Acetic Acid) until the bands appeared. Proteolytic activity was indicated as clear bands against the black background of stained gel[9].

Histone Modification Multiplex Assay

MDA-MB-231 cells were treated with TGF-β, TNF-α or TGF-β/ TNF-α and were harvested after 6 h incubation. Total Histone extracts were prepared using the EpiQuick Total Histone Extraction kit (EpigenTek, Farmingdale, NY, USA) as per the manufacturer's instructions. The concentration of the histone extracts was measured by QuickStart Bradford Dye Reagent, 1x Protein Assay Kit (Bio-Rad Laboratories, Inc, CA). Histone H3 modifications triggered by TGF-β/TNF-α treatment were then screened in 100 ng of total histone extract per well of the assay plate using Histone H3 Modification Multiplex Assay Kit (Abcam, Cambridge, UK) following the manufacturer's instructions.

Chromatin Immunoprecipitation

Chromatin immunoprecipitation (ChIP) was performed using the SimpleChIP enzymatic ChIP kit (catalog no. 9003, CST) following the manufacturer's instructions. Briefly, MDA-MB-231 cells stimulated with TGFβ and TNF-α were crosslinked with formaldehyde and digested with micrococcal nuclease followed by sonication to yield fragments ranging from 200 to 800 bp using a Covaries system. The digested chromatin fragments were subjected to immunoprecipitation using primary Abs specific to H3K36Me2 (catalog no. ab9049, Abcam), histone H3 (positive IP control, catalog no. 4620, CST), and normal rabbit IgG (negative IP control, catalog no. 2729, CST), for overnight at 4◦C and incubated with protein G magnetic beads for 2 h at 4◦C. We eluted the chromatin from an Ab/protein G magnetic beads complex by incubation at 65◦C for 30 min and by magnetic separation. We then reverse crosslinked the chromatin by treating with Proteinase K for 2 h at 65◦C and purified DNA from the ChIP fraction using the spin column method. The enrichment of DNA sequences was then detected by real-time quantitative PCR (qPCR) using SYBR Green mix and EpiTect ChIP-qPCR primers (GPH1008476(-)05A, GPH1008476(-)04A, GPH1008476(-)01A, Qiagen) spanning the MMP-9 gene promoter region.

Statistical Analysis

Statistical analyses were performed using GraphPad Prism software (version 6.07, La Jolla, CA, USA). Data are shown as ± standard error of the mean (SEM), unless otherwise indicated. Student T Test and One-way ANOVA followed by Tukey's test were used to compare means between groups. For all analyses, data from a minimum of three sample sets were used for statistical calculation, p-value <0.05 was considered significant. No significance (ns), (*$p < 0.05$)[10].

References

1. Kochumon S, Arefanian H, Sindhu S, Thomas R, Jacob T, Al-Sayyar A, Shenouda S, Al-Rashed F, Koistinen HA, Al-Mulla F *et al*: **Expression of Steroid Receptor RNA Activator 1 (SRA1) in the Adipose Tissue Is Associated with TLRs and IRFs in Diabesity**. *Cells* 2022, **11**(24).
2. Al-Mulla F, Bitar MS, Thiery JP, Zea TT, Chatterjee D, Bennett L, Park S, Edwards J, Yeung KC: **Clinical implications for loss or diminution of expression of Raf-1 kinase inhibitory protein and its phosphorylated form in ductal breast cancer**. *American journal of cancer research* 2013, **3**(5):446-464.
3. Ahmad R, Al-Roub A, Kochumon S, Akther N, Thomas R, Kumari M, Koshy MS, Tiss A, Hannun YA, Tuomilehto J *et al*: **The Synergy between Palmitate and TNF-α for CCL2 Production Is Dependent on the TRIF/IRF3 Pathway: Implications for Metabolic Inflammation**. *J Immunol* 2018, **200**(10):3599-3611.
4. Al-Rashed F, Ahmad Z, Iskandar MA, Tuomilehto J, Al-Mulla F, Ahmad R: **TNF-α Induces a Pro-Inflammatory Phenotypic Shift in Monocytes through ACSL1: Relevance to Metabolic Inflammation**. *Cell Physiol Biochem* 2019, **52**(3):397-407.
5. Kochumon S, Jacob T, Koshy M, Al-Rashed F, Sindhu S, Al-Ozairi E, Al-Mulla F, Rosen ED, Ahmad R: **Palmitate Potentiates Lipopolysaccharide-Induced IL-6 Production via Coordinated Acetylation of H3K9/H3K18, p300, and RNA Polymerase II**. *The Journal of Immunology* 2022, **209**(4):731-741.
6. Al-Roub A, Akhter N, Al-Rashed F, Wilson A, Alzaid F, Al-Mulla F, Sindhu S, Ahmad R: **TNFα induces matrix metalloproteinase-9 expression in monocytic cells through ACSL1/JNK/ERK/NF-kB signaling pathways**. *Sci Rep* 2023, **13**(1):14351.
7. Ahmad R, Kochumon S, Chandy B, Shenouda S, Koshy M, Hasan A, Arefanian H, Al-Mulla F, Sindhu S: **TNF-α Drives the CCL4 Expression in Human Monocytic Cells: Involvement of the SAPK/JNK and NF-κB Signaling Pathways**. *Cell Physiol Biochem* 2019, **52**(4):908-921.
8. Akhter N, Madhoun A, Arefanian H, Wilson A, Kochumon S, Thomas R, Shenouda S, Al-Mulla F, Ahmad R, Sindhu S: **Oxidative Stress Induces Expression of the Toll-Like Receptors (TLRs) 2 and 4 in the Human Peripheral Blood Mononuclear Cells: Implications for Metabolic Inflammation**. *Cell Physiol Biochem* 2019, **53**(1):1-18.
9. Al-Rashed F, Kochumon S, Usmani S, Sindhu S, Ahmad R: **Pam3CSK4 Induces MMP-9 Expression in Human Monocytic THP-1 Cells**. *Cell Physiol Biochem* 2017, **41**(5):1993-2003.
10. Al-Rashed F, Haddad D, Al Madhoun A, Sindhu S, Jacob T, Kochumon S, Obeid LM, Al-Mulla F, Hannun YA, Ahmad R: **ACSL1 is a key regulator of inflammatory and macrophage foaming induced by short-term palmitate exposure or acute high-fat feeding**. *iScience* 2023, **26**(7):107145.
